# Prospective associations between diet quality, frailty and incident cognitive impairment in the health and retirement study

**DOI:** 10.1186/s44263-026-00347-x

**Published:** 2026-11-11

**Authors:** May A. Beydoun, Caiqi Wei, Jordan Weiss, Hind A. Beydoun, Nicole Noren Hooten, Osorio Meirelles, Jack Tsai, Tianyi Huang, Michele K. Evans, Alan B. Zonderman

**Affiliations:** 1 Laboratory of Epidemiology and Population Sciences, National Institute on Aging, National Institutes of Health, Intramural Research Program, NIA/NIH/IRP, Baltimore, MD 21224 USA; 2 Optimal Aging Institute, New York University Grossman School of Medicine, New York, NY 10016 USA; 3 Division of Precision Medicine, Department of Medicine, New York University Grossman School of Medicine, New York, NY 10016 USA; 4 VA National Center on Homelessness Among Veterans, United States (U.S.) Department of Veterans Affairs, Washington, DC 20420 USA; 5 Department of Management, Policy, and Community Health, School of Public Health, University of Texas Health Science Center at Houston, Houston, TX 77030 USA; 6 Computational Biology & Genomics Core, National Institute on Aging, National Institutes of Health, Intramural Research Program, NIA/NIH/IRP (NIA/NIH/IRP/CBGC), Baltimore, MD 21224 USA

**Keywords:** Diet, Frailty, Cognitive impairment, Aging, Longitudinal studies

## Abstract

**Background:**

Diet quality is a modifiable determinant of healthy aging, potentially influencing physical and cognitive functioning through inflammatory and metabolic pathways. Although healthier dietary patterns have been linked to reduced frailty and cognitive decline, findings remain inconsistent. The interrelationships among diet quality, physical frailty, and incident cognitive impairment, including potential modification by specific dietary components, remain poorly understood.

**Methods:**

We analyzed 1,636 cognitively normal participants in 2016 from the Health and Retirement Study, a longitudinal study of adults in the United States (U.S.), with diet assessed in 2013, frailty in 2016, and cognitive follow-up through 2022. Cognitive impairment was assessed with cognitive tests and used the Langa–Weir classification; diet quality was measured using the Healthy Eating Index–2015 (HEI-2015); and physical frailty used a modified Fried phenotype. Survey-weighted regression, Cox models, four-way decomposition, competing-risk and multistate models, and additive Bayesian network (ABN)–informed generalized structural equation models (GSEM) were applied.

**Results:**

Higher HEI-2015 scores were associated with lower frailty (β = −0.13, P < 0.001), but not with incident cognitive impairment. Frailty was associated with higher cognitive risk in minimally adjusted models (Hazard Ratio (HR) = 2.27, 95% confidence interval (CI): 1.56–3.32), with attenuation after full adjustment. Exploratory analyses identified 13 nominally significant food-item associations with composite cognitive impairment, including higher risks associated with selected dairy products, processed meats, and beverages, and an inverse association with red wine (HR = 0.77, 95% CI: 0.66–0.89). None survived FDR correction. Five dietary associations with probable dementia alone survived FDR correction but remained exploratory. Four-way decomposition provided limited evidence of dietary mediation or interaction, while sensitivity analyses supported attenuation of frailty–cognition associations.

**Conclusions:**

Higher diet quality was associated with lower physical frailty but not independently with incident cognitive impairment. Food-specific associations were heterogeneous and largely did not survive multiple-testing correction. Findings warrant replication before drawing dietary recommendations for cognitive health.

## Background

Diet quality is an important factor that may influence healthy aging, through effect on metabolic, inflammatory, and neurodegenerative processes persisting across the life span [1]. Dietary patterns that incorporate more fruits, vegetables, whole grains, legumes, and lean protein sources have been linked to reduced cardiometabolic disease risks. These patterns also promote better functional status and enhance longevity [2]. Large prospective studies and meta-analyses have reported that alignment with healthy dietary patterns were often linked to slower cognitive decline and reduced dementia risk, despite inconsistent findings [3–5].

Frailty is a clinical syndrome associated with aging, marked by reduced physiological resilience and heightened vulnerability to stressors. It strongly predicts adverse outcomes, such as disability, hospital stays, and death [6].. Frailty encompasses physical, cognitive, and psychosocial vulnerability, with emerging constructs such as cognitive frailty emphasizing the co-occurrence of physical frailty and cognitive impairment [7–9]. Evidence from longitudinal studies indicates that frailty is strongly associated with incident cognitive decline and dementia, thereby supporting its role as a marker of biological aging [10, 11]. Prospective studies and systematic reviews have shown that alignment with healthy dietary patterns like Mediterranean-style diets, is associated with lower risk of incident frailty and slower progression of frailty components [12–16]. Such findings suggest that diet can potentially influence frailty through pathways involving muscle preservation, metabolic regulation, and inflammation[12–16].

Despite these advances, only a few studies have examined associations between diet quality, frailty, and cognitive impairment in a comprehensive analysis [17]. Concurrently, research suggests that associations between diet and cognition may be driven by specific dietary foods or food groups rather than a pre-determined overall diet quality index, with heterogeneity observed across food groups and nutrient domains [18, 19]. Capturing these complex inter-relationships requires analytic approaches beyond single-pathway models.

Using data from a nationally representative longitudinal cohort of older United States (U.S.) adults, the present study examined relationships between diet quality, frailty, and incident cognitive impairment within an integrated analytic framework. Specifically, we evaluated the association between diet quality and frailty; examined their independent and joint associations with cognitive outcomes; and conducted exploratory four-way decomposition analyses of the frailty–cognition association, with diet specified as a mediator/effect modifier. Because diet was assessed before frailty, these decompositions were interpreted as analyses of association and potential effect modification rather than causal mediation. We further applied additive Bayesian networks and generalized structural equation modeling to characterize interrelationships among diet, frailty, and cognitive impairment within a multivariable network.

## Methods

### Database and study population

The Health and Retirement Study (HRS) is an ongoing, nationally representative longitudinal study of U.S. adults aged >50 years and their spouses or partners. Conducted by the University of Michigan and sponsored by the National Institute on Aging (U01AG009740), with Social Security Administration co-funding, HRS began in 1992 and uses multistage area-probability sampling with oversampling of Black and Hispanic adults. Approximately 20,000 participants are interviewed biennially, with new cohorts added periodically to maintain population representation. Interviews collect comprehensive sociodemographic, behavioral, health, functional, employment, and cognitive information [20]..

Dietary information was obtained from the 2013 Health Care and Nutrition Study (HCNS), an HRS mail survey administered from November 2013 to May 2014 to 12,418 eligible participants. The HCNS assessed health care, food purchasing and consumption, supplements, and usual dietary intake using the Harvard food-frequency questionnaire [21]. Overall, 8,073 participants completed the survey (65% response rate), with nutrient totals available for 8,035 after prespecified exclusions [21, 22]. For the present study, 2013 HCNS dietary data were linked with primarily 2012 HRS covariates, 2016 frailty and cognition, and cognitive follow-up through 2022. Eligible participants were aged ≥50 years, cognitively normal in 2016, and had complete diet, frailty, and covariate data. Of 46,858 participants in the merged HRS and Tracker files, 20,148 met initial eligibility criteria and 2,202 had available diet and frailty data. Excluding 431 with prevalent cognitive impairment and 135 with missing covariates yielded 1,636 participants. Analyses incorporated survey weights, strata, and clustering to account for the complex HRS sampling design and differential selection probabilities [20].

### Diet quality

Diet quality was assessed using the Healthy Eating Index–2015 (HEI-2015), derived from the 2013 HCNS food-frequency questionnaire, which captured usual intake during the preceding year [21, 23, 24]. Reported food and beverage frequencies were converted to daily intake units and mapped to the 13 HEI-2015 components: total vegetables, greens and beans, total and whole fruit, whole grains, dairy, total and seafood/plant proteins, fatty-acid ratio, sodium, refined grains, added sugars, and saturated fat. Components were scored using HEI-2015 metrics and standards, with higher scores reflecting greater intake of adequacy components and lower intake of moderation components. Component scores were summed to a 0–100 HEI-2015 score, with higher values indicating greater alignment with the 2015–2020 Dietary Guidelines for Americans [25, 26]. The total HEI-2015 score and its components were standardized as z-scores for regression analyses. For descriptive analyses in Table 1, HEI-2015 scores were divided into sample-specific quartiles (Q1, ≤63.80; Q2, >63.80–70.25; Q3, >70.25–76.70; and Q4, >76.70). Supplementary Material 4 additionally presented descriptive comparisons using conventional HEI-2015 categories: poor ( < 51), needs improvement (51–80), and good ( ≥ 81). These categories were used for descriptive purposes and were not interpreted as clinically validated thresholds. The continuous standardized HEI-2015 score was retained in the primary regression analyses.

**Table 1:** Baseline characteristics of participants by HEI-2015 quartiles and frailty status (HRS 2016)

Characteristic	Overall		Q1 (lowest)		Q2		Q3		Q4 (highest)		P-value
HEI-2015 quartile			≤63.80		>63.80–70.25		>70.25–76.70		>76.70		
Sample size, unweighted	1636		409*		409*		409*		409*		—
Age (years)	67.51 (0.30)		67.12 (0.43)		67.22 (0.52)		67.70 (0.49)		67.99 (0.56)		0.4400
HEI-2015 total score, 2013	70.04 (0.36)		57.27 (0.32)		66.92 (0.11)		73.53 (0.10)		82.29 (0.28)		<0.001
Frailty score, continuous, 2016	1.41 (0.04)		1.73 (0.09)		1.45 (0.08)		1.42 (0.07)		1.05 (0.08)		<0.001
Sex	__		—		—		—		—		0.0039
Male	44.1 (1.2)		53.2 (2.4)		43.9 (2.8)		39.2 (3.1)		40.1 (2.9)		__
Female	55.9 (1.2)		46.8 (2.4)		56.1 (2.8)		60.8 (3.1)		59.9 (2.9)		__
Race/ethnicity	__		—		—		—		—		0.2152
White	84.4 (1.4)		84.6 (2.6)		87.5 (1.9)		82.7 (2.6)		83.1 (2.4)		__
Non-Hispanic Black	6.9 (0.9)		5.6 (1.3)		6.8 (1.3)		8.0 (1.7)		7.3 (1.7)		__
Hispanic	5.8 (1.0)		7.8 (2.3)		4.7 (1.5)		5.7 (1.5)		5.0 (0.9)		__
Others	2.8 (0.5)		2.1 (0.9)		1.0 (0.6)		3.6 (1.1)		4.6 (1.3)		__
Total household income, 2012	__		—		—		—		—		<0.001
< $25,000	17.4 (1.1)		24.8 (2.4)		13.5 (2.0)		16.8 (2.2)		14.4 (2.4)		__
$25k–124,999	62.9 (1.5)		63.4 (2.6)		67.3 (3.1)		61.0 (3.2)		60.0 (3.0)		__
$125k–299,999	15.1 (1.5)		8.4 (1.7)		15.4 (2.3)		17.2 (2.5)		19.2 (3.4)		__
$300k–649,999	4.1 (0.9)		3.5 (1.5)		3.7 (1.4)		5.1 (1.7)		4.2 (1.5)		__
≥$650k	0.6 (0.3)		0.0 (0.0)		0.1 (0.1)		0.0 (0.0)		2.1 (1.1)		__
Marital status, 2012	__		—		—		—		—		0.1610
Never married	6.0 (0.9)		7.4 (1.6)		7.7 (2.0)		4.0 (1.4)		4.9 (1.3)		__
Married/partnered	71.1 (1.4)		65.0 (3.2)		73.9 (2.5)		74.7 (2.2)		71.0 (2.8)		__
Separated/Divorced	14.0 (1.2)		16.4 (2.0)		10.9 (2.3)		14.6 (1.8)		14.1 (2.4)		__
Widowed	8.9 (0.6)		11.2 (1.8)		7.5 (1.4)		6.7 (1.4)		10.0 (1.8)		__
Educational attainment	__		—		—		—		—		0.0027
Less than high school	6.4 (0.7)		10.2 (1.7)		3.9 (1.0)		6.4 (1.4)		4.8 (1.0)		__
GED	4.8 (0.6)		4.7 (1.3)		4.8 (1.4)		4.9 (1.5)		4.9 (1.4)		__
High School graduate	27.0 (1.4)		31.9 (2.5)		26.9 (2.7)		28.7 (2.2)		20.8 (1.9)		__
Some college	28.5 (1.5)		30.0 (2.9)		31.6 (3.7)		25.2 (3.0)		27.3 (2.6)		__
College degree or higher	33.3 (1.8)		23.2 (2.9)		32.9 (3.2)		34.8 (2.6)		42.2 (3.4)		__
Smoking status, 2012	__		—		—		—		—		0.0011
Never	47.8 (1.6)		41.8 (3.0)		44.1 (3.2)		51.1 (3.2)		54.3 (3.1)		__
Former	41.1 (1.6)		40.6 (2.9)		43.1 (3.0)		39.5 (2.7)		41.3 (3.1)		__
Current	11.1 (0.8)		17.7 (2.8)		12.9 (1.8)		9.4 (1.9)		4.4 (1.6)		__
Alcohol consumption, 2012	__		—		—		—		—		0.8297
Abstinent	37.7 (1.7)		42.4 (2.8)		37.1 (3.6)		35.6 (3.2)		35.5 (3.0)		__
1-3 days per month	19.8 (1.3)		19.4 (2.5)		17.8 (2.3)		20.7 (2.6)		21.2 (2.3)		__
1-2 days per week	24.6 (1.3)		21.8 (2.2)		26.5 (3.2)		25.4 (3.0)		24.9 (2.7)		__
≥3 days per week	17.9 (1.3)		16.4 (2.0)		18.6 (2.0)		18.3 (3.1)		18.4 (2.7)		__
Physical activity, 2012	__		—		—		—		—		<0.001
Low	12.1 (0.8)		20.0 (2.3)		11.9 (2.1)		10.5 (1.7)		5.9 (1.3)		__
Medium	26.4 (1.3)		32.5 (2.8)		27.3 (2.5)		27.6 (2.6)		18.6 (2.5)		__
High	61.5 (1.5)		47.5 (3.3)		60.8 (3.4)		62.0 (2.9)		75.5 (2.4)		__
Self-rated health, 2012	__		—		—		—		—		0.0077
Good/Excellent	83.1 (1.0)		77.9 (2.8)		85.7 (2.0)		80.5 (2.5)		88.3 (1.8)		__
Fair/Poor	16.9 (1.0)		22.1 (2.8)		14.3 (2.0)		19.6 (2.5)		11.7 (1.8)		__
Body Mass Index, categorical, 2012	__		—		—		—		—		0.1056
Normal	25.5 (1.1)		21.5 (1.6)		22.8 (2.6)		24.5 (2.6)		33.2 (3.2)		__
Overweight	35.7 (1.3)		36.4 (3.4)		37.8 (3.3)		35.2 (3.1)		33.7 (2.9)		__
Obese	38.7 (1.9)		42.1 (3.6)		39.4 (3.4)		40.4 (3.5)		33.1 (3.5)		__
High blood pressure, 2012	__		—		—		—		—		0.2521
No	50.8 (1.6)		45.8 (3.1)		49.4 (3.1)		54.2 (3.4)		53.6 (3.6)		__
Yes	49.2 (1.6)		54.2 (3.1)		50.6 (3.1)		45.8 (3.4)		46.4 (3.6)		__
Diabetes, 2012	__		—		—		—		—		0.7279
No	84.1 (1.4)		83.4 (2.6)		84.0 (2.1)		86.3 (2.1)		82.9 (2.8)		__
Yes	15.9 (1.4)		16.6 (2.6)		15.9 (2.1)		13.7 (2.1)		17.1 (2.8)		__
Stroke, 2012	__		—		—		—		—		0.3082
No	94.9 (0.6)		96.7 (0.9)		93.9 (1.3)		94.1 (1.3)		94.9 (1.3)		__
Yes	5.1 (0.6)		3.3 (0.9)		6.1 (1.3)		5.9 (1.3)		5.1 (1.3)		__
Heart disease, 2012	__		—		—		—		—		0.9054
No	82.0 (1.3)		80.9 (2.8)		83.2 (2.4)		81.7 (2.1)		82.3 (2.0)		__
Yes	18.0 (1.3)		19.1 (2.8)		16.8 (2.4)		18.3 (2.1)		17.7 (2.0)		__
Frailty status, 2016	__		—		—		—		—		<0.001
Robust	24.4 (1.5)		15.4 (2.8)		21.9 (2.9)		21.8 (2.3)		38.3 (3.5)		__
Pre-frail	57.2 (1.5)		58.3 (3.0)		59.8 (2.8)		61.2 (2.4)		49.7 (2.8)		__
Frail	18.4 (1.3)		26.2 (3.0)		18.4 (2.2)		17.0 (2.2)		12.0 (2.0)		__
Characteristic		**Overall**		**Non-frail**		**Pre-frail**		**Frail**		**P-value**	
Sample size, unweighted		1636		341		959		336		__	
Age (years)		67.51 (0.30)		65.58 (0.42)		67.10 (0.34)		71.35 (0.65)		<0.001	
HEI-2015 total score, 2013		70.04 (0.36)		73.57 (0.77)		69.44 (0.42)		67.22 (0.73)		<0.001	
Frailty score, continuous, 2016		1.41 (0.04)		0.00 (0.00)		1.41 (0.02)		3.29 (0.04)		<0.001	
Sex		__		__		__		__		<0.001	
Male		44.1 (1.2)		54.5 (3.1)		41.5 (1.8)		38.5 (2.9)		__	
Female		55.9 (1.2)		45.5 (3.1)		58.5 (1.8)		61.5 (2.9)		__	
Race/ethnicity		__		__		__		__		0.1365	
White		84.4 (1.4)		88.8 (2.2)		83.4 (1.6)		82.0 (3.3)		__	
Non-Hispanic Black		6.9 (0.9)		4.6 (1.1)		7.7 (1.2)		7.6 (1.7)		__	
Hispanic		5.8 (1.0)		4.2 (1.2)		5.4 (1.0)		9.0 (3.1)		__	
Others		2.8 (0.5)		2.4 (1.2)		3.5 (0.6)		1.4 (0.9)		__	
Total household income, 2012		__		__		__		__		<0.001	
< $25,000		17.4 (1.1)		7.2 (1.2)		14.9 (1.2)		38.7 (3.4)		__	
$25k–124,999		62.9 (1.5)		56.9 (3.3)		67.6 (1.6)		56.1 (3.4)		__	
$125k–299,999		15.1 (1.5)		26.9 (3.2)		13.5 (1.7)		4.0 (1.3)		__	
$300k–649,999		4.1 (0.9)		7.1 (2.2)		3.9 (1.0)		0.8 (0.6)		__	
≥$650k		0.6 (0.3)		2.0 (1.1)		0.1 (0.1)		0.3 (0.3)		__	
Marital status, 2012		__		__		__		__		<0.001	
Never married		6.0 (0.9)		4.0 (1.4)		5.3 (1.2)		10.9 (2.2)		__	
Married/partnered		71.1 (1.4)		81.8 (2.3)		70.1 (1.9)		59.9 (3.7)		__	
Separated/Divorced		14.0 (1.2)		10.0 (2.4)		15.9 (1.5)		13.4 (2.5)		__	
Widowed		8.9 (0.6)		4.1 (1.2)		8.7 (0.8)		15.8 (2.0)		__	
Educational attainment		__		__		__		__		<0.001	
Less than high school		6.4 (0.7)		2.9 (0.7)		5.4 (1.0)		13.9 (2.8)		__	
GED		4.8 (0.6)		2.3 (0.9)		4.7 (0.9)		8.4 (2.1)		__	
High School graduate		27.0 (1.4)		17.2 (2.6)		28.1 (1.8)		36.7 (3.3)		__	
Some college		28.5 (1.5)		27.3 (2.9)		29.2 (1.7)		28.1 (3.0)		__	
College degree or higher		33.3 (1.8)		50.3 (3.8)		32.5 (2.2)		12.9 (2.5)		__	
Smoking status, 2012		__		__		__		__		0.0028	
Never		47.8 (1.6)		53.5 (3.0)		48.9 (2.2)		36.8 (3.7)		__	
Former		41.1 (1.6)		38.8 (3.0)		40.6 (2.1)		45.7 (3.4)		__	
Current		11.1 (0.8)		7.7 (1.7)		10.4 (1.2)		17.5 (2.3)		__	
Alcohol consumption, 2012		__		__		__		__		<0.001	
Abstinent		37.7 (1.7)		26.0 (2.7)		37.7 (2.3)		53.0 (2.9)		__	
1-3 days per month		19.8 (1.3)		22.6 (2.2)		18.3 (1.8)		20.4 (3.0)		__	
1-2 days per week		24.6 (1.3)		26.1 (2.5)		27.2 (2.0)		14.6 (2.2)		__	
≥3 days per week		17.9 (1.3)		25.3 (2.7)		16.7 (1.6)		11.9 (2.3)		__	
Physical activity, 2012		__		__		__		__		<0.001	
Low		12.1 (0.8)		1.0 (0.6)		12.0 (1.3)		27.0 (2.5)		__	
Medium		26.4 (1.3)		13.9 (2.1)		28.8 (1.6)		35.8 (3.1)		__	
High		61.5 (1.5)		85.1 (2.1)		59.2 (1.8)		37.2 (2.6)		__	
Self-rated health, 2012		__		__		__		__		<0.001	
Good/Excellent		83.1 (1.0)		95.7 (1.2)		86.0 (1.0)		57.6 (4.0)		__	
Fair/Poor		16.9 (1.0)		4.3 (1.2)		14.0 (1.0)		42.4 (4.0)		__	
Body Mass Index, categorical, 2012		__		__		__		__		<0.001	
Normal		25.5 (1.1)		37.5 (3.2)		22.0 (1.3)		20.8 (2.6)		__	
Overweight		35.7 (1.3)		40.2 (3.0)		36.6 (1.7)		27.1 (3.0)		__	
Obese		38.7 (1.9)		22.3 (2.5)		41.4 (2.3)		52.1 (3.7)		__	
High blood pressure, 2012		__		__		__		__		<0.001	
No		50.8 (1.6)		59.1 (2.7)		51.8 (2.1)		36.6 (3.2)		__	
Yes		49.2 (1.6)		40.9 (2.7)		48.2 (2.1)		63.4 (3.2)		__	
Diabetes, 2012		__		__		__		__		<0.001	
No		84.1 (1.4)		89.2 (2.0)		85.5 (1.6)		73.1 (2.9)		__	
Yes		15.9 (1.4)		10.8 (2.0)		14.5 (1.6)		26.9 (2.9)		__	
Stroke, 2012		__		__		__		__		0.0175	
No		94.9 (0.6)		98.3 (0.9)		94.2 (0.7)		92.6 (1.8)		__	
Yes		5.1 (0.6)		1.7 (0.9)		5.8 (0.7)		7.4 (1.8)		__	
Heart disease, 2012		__		__		__		__		<0.001	
No		82.0 (1.3)		86.9 (2.5)		83.7 (1.4)		70.0 (3.0)		__	
Yes		18.0 (1.3)		13.1 (2.5)		16.3 (1.4)		30.0 (3.0)		__	
Frailty status, 2016		__		__		__		__		__	
Robust		24.4 (1.5)		100.0 (0.0)		0.0 (0.0)		0.0 (0.0)		__	
Pre-frail		57.2 (1.5)		0.0 (0.0)		100.0 (0.0)		0.0 (0.0)		__	
Frail		18.4 (1.3)		0.0 (0.0)		0.0 (0.0)		100.0 (0.0)		__	

*BMI* body mass index, *GED* general educational development, *HEI-2015* healthy eating index–2015, *HRS* health and retirement study, *SE* standard error

Data are from the Health and Retirement Study (HRS), a nationally representative longitudinal cohort of U.S. adults aged ≥50 years, linking 2013 Health Care and Nutrition Study dietary data, 2012 covariates, baseline 2016 frailty and cognition, and follow-up through 2022. The analytic sample includes 1,636 participants who were cognitively normal at baseline and had complete data on diet, frailty, and covariates

Values are survey-weighted means (standard errors) for continuous variables and percentages (standard errors) for categorical variables. P-values are based on design-adjusted Wald tests for continuous variables and Rao–Scott χ² tests for categorical variables. HEI-2015 categories were defined using quartiles within final selected sample. Frailty score represents the sum of five components based on a modified Fried phenotype

Exploratory analyses examined standardized food items across seven HCNS food groups, identified by food-group codes C3–C9: C3, dairy, alternatives, and spreads/fats; C4, fruits and juices; C5, vegetables, legumes, and soy; C6, eggs, meats, seafood, and other proteins; C7, grains, breads, starches, and savory snacks; C8, beverages and alcohol; and C9, sweets, snacks, condiments, and added fats. Complete item definitions, units, and scoring rules are provided in Supplementary Materials 3: Supplementary Datasheet 1.

### Frailty

Frailty was assessed in 2016 using a modified Fried phenotype comprising five binary components: shrinking, weakness, exhaustion, slowness, and low physical activity [27–29]. Weakness was performance-based and defined as grip strength in the lowest sex-specific quintile ( ≤ 27.125 kg for men and ≤16.625 kg for women). The remaining components were derived from self-reported information or reported measures: shrinking was defined by reported weight loss or a body mass index <18.5 kg/m²; exhaustion by endorsement of either of two fatigue items from the Center for Epidemiologic Studies Depression scale (CES-D) [30]; slowness by reported difficulty walking several blocks; and low physical activity by participation in moderate or vigorous activity no more than one to three times per month. Each component was coded as present or absent, and all five components were required to calculate the total score. Components were summed to produce a frailty score ranging from 0 to 5, with participants classified as robust (0), pre-frail (1–2), or frail ( ≥ 3). Frailty was examined as both a continuous score and a categorical measure. Additional operational details are provided in Supplementary Materials 1: Appendix A, while supplementary methods and results are presented additionally in Supplementary Materials 1:Appendices B-H.

### Cognitive impairment

Incident cognitive impairment was defined using the Langa–Weir classification, which categorizes participants as cognitively normal, cognitively impaired non-dementia (CIND), or having probable dementia based on cognitive testing and proxy assessments [31, 32]. Participants were required to be cognitively normal at baseline in 2016 and were evaluated at three biennial follow-up assessments in 2018, 2020, and 2022. Follow-up began at baseline, with incident events assigned to the midpoint between the preceding assessment and the first assessment showing impairment. The primary composite outcome was time to the first observed transition from normal cognition to either CIND or probable dementia. Subsequent progression from CIND to probable dementia was not counted as another composite event and was evaluated separately in the multistate analysis described in Supplementary Materials 1: Appendix E. In the primary Cox models, participants without an event were censored at the earliest of death, their last observed cognitively normal assessment, or the administrative end of follow-up on December 31, 2022. CIND-only and probable-dementia-only outcomes were also examined. Death was treated as a competing event in Fine–Gray analyses and as a separate absorbing state in multistate analyses.

### Covariates

Covariates were selected a priori based on established determinants of diet, frailty, and cognitive outcomes. Age was measured in 2016 and modeled continuously; sex and race/ethnicity were obtained from cross-wave HRS variables. Race/ethnicity was categorized as non-Hispanic White, non-Hispanic Black, Hispanic of any race, or non-Hispanic other race. The remaining covariates were measured in 2012 [20]. Socioeconomic characteristics included education (less than high school, General Educational Development credential, high-school graduate, some college, or college degree or higher), total household income (<$25,000, $25,000–$124,999, $125,000–$299,999, $300,000–$649,999, or ≥$650,000), and marital status (never married, married or partnered, separated or divorced, or widowed). Lifestyle factors included smoking status (never, former, or current), alcohol use (abstinent, 1–3 days/month, 1–2 days/week, or ≥3 days/week), and physical activity based on the highest reported frequency of moderate or vigorous activity (hardly ever or never, 1–3 times/month to once/week, or more than once/week to daily). Clinical covariates included body mass index (Body Mass Index, BMI; <25, 25–29.9, or ≥30 kg/m²), self-reported hypertension, diabetes, stroke, and heart disease, and self-rated health (excellent, very good, or good versus fair or poor). BMI was preferentially obtained from the HRS physical-measures variable and supplemented, when unavailable, using the harmonized BMI variable from the RAND HRS longitudinal file. The measurement wave and any longitudinal carry-forward procedures were determined according to the underlying variable construction and analytic data-processing procedures. Values ≥ 100 kg/m² were treated as implausible and set to missing; no lower exclusion threshold was applied, and therefore the lowest category included all eligible BMI values < 25 kg/m². In network and structural-equation analyses, socioeconomic status was additionally summarized as a standardized principal-component factor derived from education and household income.

### Statistical analysis

Analyses accounted for the complex HRS sampling design using the HCNS sampling weight, stratification variable, and primary sampling unit. Variances and standard errors were estimated using first-order Taylor-series linearization, with single-unit strata centered at the overall mean [33]. Baseline characteristics were summarized using survey-weighted means and standard errors for continuous variables and weighted proportions and standard errors for categorical variables. Group differences by HEI-2015 quartiles and frailty status in the primary descriptive analysis, and by conventional HEI-2015 categories in sensitivity analyses, were evaluated using design-adjusted Wald tests for continuous variables and Rao–Scott χ² tests for categorical variables.

For regression analyses, continuous dietary variables—including the HEI-2015 total score, its 13 component scores, and individual food-group variables—were converted to z-scores within the final analytic sample as $z=(x-\bar{x})/{SD}$, using the unweighted analytic-sample mean and standard deviation. Standardization placed dietary measures with different units and ranges on a common scale; coefficients and hazard ratios therefore represent the association with a 1-SD higher dietary value. Associations between diet quality and frailty were examined using survey-weighted linear regression, as follows:

${\mathrm{Frailty}}_{i}={\beta }_{0}+{\beta }_{1}{\mathrm{HEI}}_{i}+{{\bf{X}}}_{i}^{{\rm{\top }}}{\boldsymbol{\beta }}+{\varepsilon }_{i}$, where HEI-2015 was z-score standardized and ${{\bf{X}}}_{i}$ included demographic, socioeconomic, lifestyle, and clinical covariates.

Models were built sequentially. Model 1 adjusted for age and sex. Model 2 additionally adjusted for race/ethnicity, marital status, education, and household income. Model 3 further adjusted for smoking status, alcohol use, physical activity, body mass index, hypertension, diabetes, stroke, heart disease, and self-rated health. In analyses of frailty in relation to cognitive outcomes, Model 3 also adjusted for HEI-2015 to estimate the association of frailty with cognition independently of overall diet quality. Model 3 was prespecified as the primary model for inferential interpretation because it provided the most comprehensive adjustment for measured sociodemographic, behavioral, and clinical factors potentially associated with both frailty and cognitive outcomes. Models 1 and 2 were retained to demonstrate attenuation with sequential adjustment and were considered supporting analyses.

Prospective associations with incident cognitive impairment were evaluated using survey-weighted Cox proportional hazards models:

$${h}_{i}(a{\rm{| }}{F}_{i},{{\bf{X}}}_{{im}})={h}_{0}(a)\exp \left\{{\beta }_{F}{F}_{i}+{{\boldsymbol{\beta }}}_{m}^{T}{{\bf{X}}}_{{im}}\right\},a\ge {a}_{i0},$$

where *a* is attained age, ${a}_{i0}$ is age at study entry, ${F}_{i}$ represents continuous or categorical frailty, and ${{\bf{X}}}_{{im}}$ represents the covariates included in adjustment Model *m*. Age was used as the underlying time scale, with delayed entry at baseline age to account for left truncation. Outcomes included composite cognitive impairment (CIND or probable dementia), CIND-only, and probable-dementia-only endpoints.

Because analyses of HEI-2015 components and individual food items were exploratory and involved multiple comparisons, nominal P values were supplemented with Benjamini–Hochberg false discovery rate (FDR)-adjusted q values. FDR correction was applied separately within each cognitive outcome and covariate-adjustment model across the dietary tests reported in Supplementary Materials 3: Supplementary Datasheet 3. Associations with q < 0.05 were considered to survive FDR correction, whereas nominal associations not meeting this threshold were interpreted as exploratory signals.

To assess departures from linearity, graded associations, and potential threshold effects, key exposures were also modeled categorically in secondary analyses. HEI-2015 was divided into sample-specific quartiles, with the lowest quartile as the reference, whereas frailty was classified as robust (0 components), pre-frail (1–2 components), or frail ( ≥ 3 components), with robust participants as the reference. Hazard ratios and 95% confidence intervals were estimated for each category, and overall associations were evaluated using design-adjusted Wald tests. Trends were assessed by modeling the ordered HEI-2015 quartiles or frailty categories as continuous ordinal variables. These categories were used to characterize the shape of the associations and were not interpreted as validated clinical thresholds. These reference categories represented the lowest ordered level of each exposure: Q1 allowed comparison with progressively higher HEI-2015 scores, whereas robust status allowed comparison with increasing frailty. Thus, the categories were aligned statistically by scale order, although higher HEI-2015 indicates better health and higher frailty indicates poorer health.

Counterfactual four-way decomposition (med4way) was used to partition the frailty–cognition association, with frailty specified as the exposure and diet (HEI-2015 total score, components, or food groups) as the mediator/effect modifier, restricted to frailty–cognition associations significant at *P* < 0.05 [34]. Because diet was assessed before frailty, these analyses were considered exploratory decompositions of the frailty–cognition association and potential frailty–diet interaction rather than causal mediation analyses. The total effect of frailty on incident cognitive impairment was thereby partitioned into controlled direct, reference interaction, mediated interaction, and pure indirect effect under a Cox proportional hazards outcome model and linear mediator model. Specifically, the mediator model was defined as:

$${M}_{i}={\alpha }_{0}+{\alpha }_{1}{{\rm{Frailty}}}_{i}+{{\rm{X}}}_{i}^{{\rm{\top }}}\alpha +{\epsilon }_{i},$$

where ${M}_{i}$ represents standardized HEI-2015 total score, component scores or dietary food groups. The outcome model included main effects for frailty and diet, as well as their interaction term, allowing assessment of whether the association between frailty and cognitive impairment varies across levels of diet quality [34].

The four components of the total effect were interpreted as follows: the pure natural direct effect (PNDE) represents the effect of frailty on cognitive impairment not operating through diet; the pure natural indirect effect (PNIE) represents mediation through diet in the absence of interaction; the reference interaction (INTref) captures effect modification by diet independent of mediation; and the mediated interaction (INTmed) reflects the joint contribution of mediation and interaction [34]. The sum of PNIE and INTmed represents overall mediated effect, while INTref and INTmed together quantify the extent to which diet modifies the effect of frailty on cognition [34].

Additive Bayesian networks (ABN) were used to identify conditional dependency structures among diet, frailty, socioeconomic factors, and cognition [35–38]. The joint distribution was factorized as $P({\bf{V}})={\prod }_{j}P({V}_{j}| \mathrm{Pa}({V}_{j}))$ model selection used the Bayesian Information Criterion under temporal-ordering constraints and parent-count limits to reduce overfitting. The ABN search space was constrained a priori according to temporal and substantive considerations by identifying variables that were exogenous to the system and variables permitted to directly predict the final outcome, incident Mild Cognitive Impairment (MCI)/dementia. Diet quality, measured in 2013, was modeled as an upstream lifestyle factor and was allowed to predict other lifestyle and health-related factors, frailty measured in 2016, and incident MCI/dementia.

Paths identified by ABN were subsequently quantified using generalized structural equation models, allowing simultaneous estimation of multiple pathways. Time-to-event outcomes were modeled using a Weibull specification, $h(t)=\lambda p{t}^{p-1}\exp (\eta )$, with exponentiated coefficients interpreted as hazard ratios. Sensitivity analyses included additional lifestyle and energy-intake adjustment, alternative frailty parameterizations, and Schoenfeld-residual checks of proportional hazards.

In a series of sensitivity analyses, we assessed robustness using competing risks and multistate models. Competing risks regression accounted for death as a competing event, estimating sub-distribution hazards while retaining individuals who died in the risk set. Multistate analyses considered transitions among normal cognition, MCI/CIND, probable dementia, and death; transition-specific Weibull models were estimated only for transitions with ≥40 events. A reduced ABN was then constructed by removing time indicators and retaining key pathways. This structure informed a generalized structural equation model (GSEM), which simultaneously estimated linked equations for all variables. Continuous and categorical variables were modeled with appropriate links, and incident composite cognitive impairment was modeled as a parametric survival outcome. Robust standard errors accounted for clustering. This framework enabled estimation of direct and indirect pathways while preserving the ABN-derived dependency structure. Most statistical analyses were conducted using Stata version 19.0 (StataCorp LLC, College Station, TX, USA), whereas ABN analyses and data visualizations were performed using R (version 4.6.0; R Foundation for Statistical Computing, Vienna, Austria).

## Results

### Study size and baseline characteristics

**Fig. 1:**
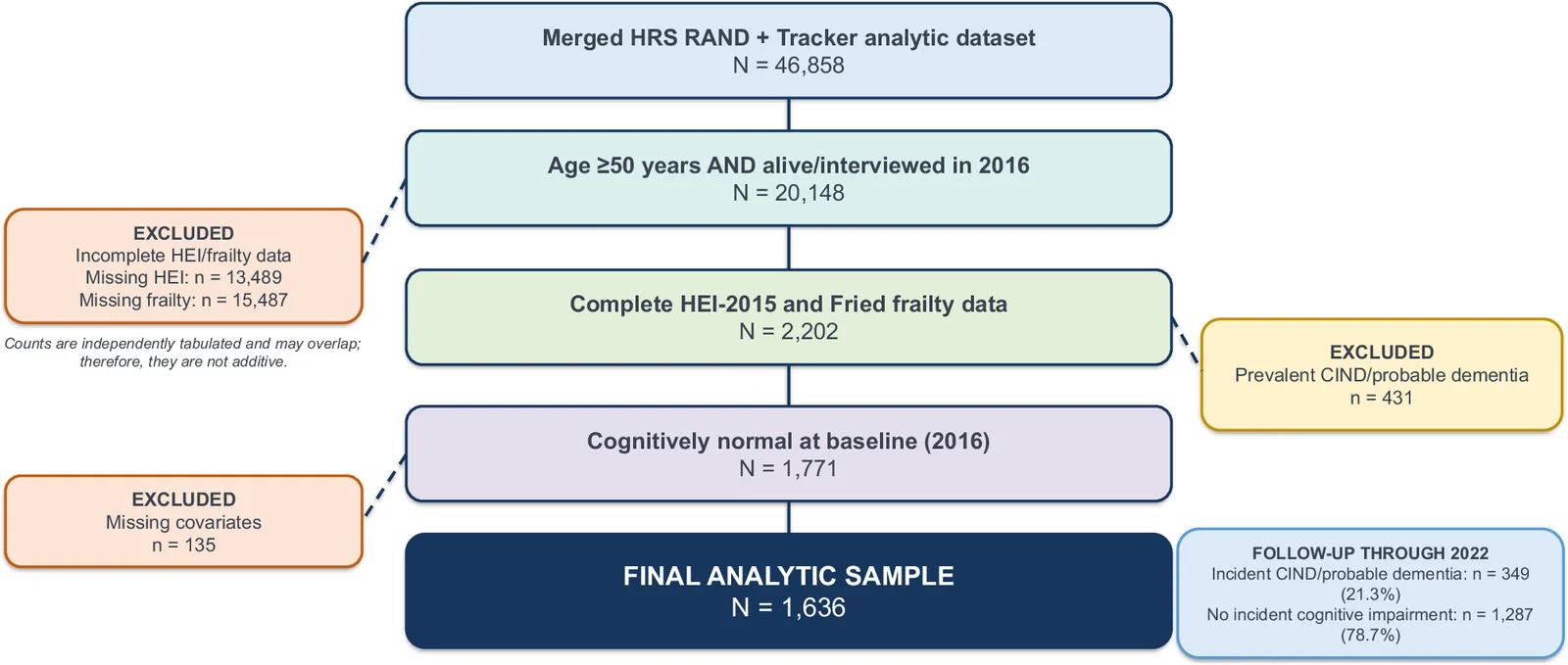
Participant flow diagram of the analytic sample (Health and Retirement Study). Participant selection from the Health and Retirement Study is shown. Of 46,858 participants in the merged RAND and Tracker files, 20,148 were aged ≥50 years, alive, and interviewed during the study period. Exclusions for unavailable dietary or frailty data yielded 2,202 participants with both measures. After excluding 431 participants with baseline cognitive impairment and 135 with missing covariates, the final analytic sample included 1,636 cognitively normal participants. HRS = Health and Retirement Study; HEI-2015 = Healthy Eating Index–2015; RAND = RAND HRS longitudinal file

The analytic sample was derived by linking covariates obtained primarily in 2012, dietary data collected in 2013, frailty and baseline cognition assessed in 2016, and cognitive follow-up conducted during 2018–2022 (Fig. 1). Among 46,858 participants, 20,148 were eligible. After exclusions for missing diet or frailty, 2,202 remained; excluding 431 with prevalent cognitive impairment and 135 with missing covariates yielded a final sample of 1,636 participants.

Table 1 presents baseline characteristics by HEI-2015 quartiles and frailty status. Higher HEI-2015 quartiles were associated with progressively lower mean frailty scores (1.73 in Q1 versus 1.05 in Q4; P < 0.001) and a lower prevalence of frailty (26.2% versus 12.0%; P < 0.001). Higher diet quality was also associated with greater educational attainment (P = 0.0027), higher household income (P < 0.001), lower current smoking prevalence (P = 0.0011), greater physical activity (P < 0.001), and better self-rated health (P = 0.0077). Sex distribution differed across quartiles (P = 0.0039), whereas age, race/ethnicity, marital status, alcohol consumption, body mass index, and chronic conditions did not differ significantly. Conversely, frail participants were older (71.4 versus 65.6 years), had lower mean HEI-2015 scores (67.2 versus 73.6), and exhibited greater socioeconomic, behavioral, and clinical vulnerability than robust participants.

Supplementary Materials 4 provides expanded survey-weighted descriptive characteristics by conventional HEI-2015 categories and frailty status, including individual HEI-2015 components and food-frequency items. Higher HEI-2015 categories were associated with higher component scores across multiple dietary domains, including fruits, vegetables, whole grains, and moderation components such as sodium and added sugars. Comparisons by frailty status also revealed differences across several dietary components, although associations were heterogeneous. These descriptive findings complement the quartile-based comparisons in Table 1 and suggest that differences in overall diet quality reflect variation across multiple dietary components rather than a single food group.

### Primary analyses

Table 2 shows selected survey-weighted multivariable linear regression models examining the association between diet quality and frailty score. Higher HEI-2015 scores were consistently associated with lower frailty across all models. In minimally adjusted analyses, each 1-SD increase in HEI was associated with a 0.27-unit lower frailty score (β = −0.27, P < 0.001), with attenuation but persistence of the association after adjustment for socioeconomic, behavioral, and clinical factors (fully adjusted β = −0.13, P < 0.001). Analyses of individual HEI components showed broadly consistent directions, with healthier dietary components (e.g., vegetables) associated with lower frailty, supporting the interpretation that overall dietary pattern quality, rather than a single component, drives the observed association. The full set of results is presented in Supplementary Materials 3: Supplementary Datasheet 2.

**Table 2:** Association of HEI-2015 total score, HEI-2015 components, and selected food-group variables with frailty score in the health and retirement study (n = 1,636)

Exposure	Model 1 β (SE), p	Model 2 β (SE), p	Model 3 β (SE), p
HEI-2015 total score and component scores			
HEI-2015 total score	-0.268 (0.043), <0.001	-0.197 (0.035), <0.001	-0.133 (0.034), <0.001
Total vegetables	-0.181 (0.034), <0.001	-0.103 (0.034), 0.004	-0.067 (0.031), 0.033
Greens and beans	-0.181 (0.043), <0.001	-0.121 (0.034), 0.001	-0.088 (0.027), 0.002
Total fruit	-0.182 (0.032), <0.001	-0.142 (0.026), <0.001	-0.098 (0.029), 0.001
Whole fruit	-0.152 (0.040), <0.001	-0.119 (0.037), 0.002	-0.073 (0.033), 0.034
Whole grains	-0.089 (0.037), 0.022	-0.070 (0.027), 0.012	-0.063 (0.028), 0.030
Dairy	-0.045 (0.039), 0.252	-0.045 (0.034), 0.185	-0.036 (0.029), 0.218
Total protein foods	-0.099 (0.037), 0.009	-0.075 (0.034), 0.030	-0.084 (0.030), 0.008
Seafood and plant proteins	-0.177 (0.040), <0.001	-0.120 (0.036), 0.002	-0.091 (0.032), 0.006
Fatty acid ratio	-0.151 (0.043), 0.001	-0.101 (0.038), 0.010	-0.054 (0.033), 0.108
Sodium	-0.103 (0.040), 0.013	-0.084 (0.037), 0.028	-0.046 (0.033), 0.171
Refined grains	-0.106 (0.053), 0.052	-0.043 (0.044), 0.331	-0.027 (0.039), 0.495
Added sugar	-0.039 (0.021), 0.070	-0.010 (0.017), 0.534	0.023 (0.020), 0.262
Saturated fat	-0.096 (0.039), 0.018	-0.075 (0.034), 0.033	-0.018 (0.035), 0.612
Food-group variables: Dairy, dairy alternatives, and spreads/fats			
C3A_FF_13: Skim Milk [Dairy, dairy alternatives, and spreads/fats]	-0.080 (0.028), 0.007	-0.050 (0.024), 0.041	-0.010 (0.022), 0.653
C3H_FF_13: Regular Ice Cream [Dairy, dairy alternatives, and spreads/fats]	0.074 (0.027), 0.008	0.064 (0.026), 0.018	0.058 (0.026), 0.028
C3J_FF_13: Low Carb Yogurt [Dairy, dairy alternatives, and spreads/fats]	-0.062 (0.023), 0.010	-0.012 (0.025), 0.632	0.010 (0.025), 0.688
C3Q_FF_13: Spreadable Butter [Dairy, dairy alternatives, and spreads/fats]	0.099 (0.038), 0.012	0.060 (0.026), 0.023	0.034 (0.019), 0.077
C3R_FF_13: Margarine [Dairy, dairy alternatives, and spreads/fats]	0.091 (0.036), 0.016	0.041 (0.033), 0.226	0.011 (0.038), 0.770
Food-group variables: Fruits and fruit juices			
C4D_FF_13: Bananas [Fruits and fruit juices]	-0.061 (0.028), 0.034	-0.079 (0.025), 0.002	-0.068 (0.024), 0.007
C4F_FF_13: Avocado [Fruits and fruit juices]	-0.134 (0.040), 0.002	-0.119 (0.033), 0.001	-0.067 (0.033), 0.047
C4G_FF_13: Applesauce [Fruits and fruit juices]	0.067 (0.033), 0.048	0.049 (0.034), 0.156	0.056 (0.028), 0.053
C4H_FF_13: Apples/Pears [Fruits and fruit juices]	-0.130 (0.026), <0.001	-0.117 (0.029), <0.001	-0.081 (0.024), 0.001
C4P_FF_13: Strawberries [Fruits and fruit juices]	-0.126 (0.033), <0.001	-0.077 (0.029), 0.011	-0.048 (0.026), 0.071
C4Q_FF_13: Other Berries [Fruits and fruit juices]	-0.212 (0.027), <0.001	-0.127 (0.025), <0.001	-0.064 (0.022), 0.005
C4S_FF_13: Apricots [Fruits and fruit juices]	-0.090 (0.026), 0.001	-0.038 (0.024), 0.110	-0.025 (0.023), 0.267
Food-group variables: Vegetables, legumes, and soy foods			
C5A_FF_13: Tomatoes [Vegetables, legumes, and soy foods]	-0.123 (0.041), 0.004	-0.091 (0.036), 0.015	-0.065 (0.033), 0.059
C5D_FF_13: Salsa [Vegetables, legumes, and soy foods]	-0.121 (0.032), <0.001	-0.117 (0.028), <0.001	-0.056 (0.031), 0.073
C5E_FF_13: Tofu Soy Protein [Vegetables, legumes, and soy foods]	-0.126 (0.027), <0.001	-0.076 (0.026), 0.005	-0.053 (0.029), 0.067
C5G_FF_13: Broccoli [Vegetables, legumes, and soy foods]	-0.113 (0.035), 0.002	-0.076 (0.029), 0.010	-0.041 (0.023), 0.085
C5I_FF_13: Cauliflower [Vegetables, legumes, and soy foods]	-0.053 (0.026), 0.048	-0.041 (0.027), 0.130	-0.020 (0.026), 0.449
C5J_FF_13: Brussels Sprouts [Vegetables, legumes, and soy foods]	-0.091 (0.040), 0.025	-0.070 (0.023), 0.004	-0.046 (0.024), 0.056
C5K_FF_13: Carrots Raw [Vegetables, legumes, and soy foods]	-0.180 (0.031), <0.001	-0.142 (0.023), <0.001	-0.089 (0.020), <0.001
C5O_FF_13: Mixed Vegetables [Vegetables, legumes, and soy foods]	-0.084 (0.029), 0.006	-0.067 (0.026), 0.011	-0.020 (0.023), 0.383
C5P_FF_13: Beans Or Lentils [Vegetables, legumes, and soy foods]	-0.070 (0.032), 0.032	-0.069 (0.031), 0.029	-0.051 (0.028), 0.069
C5Q_FF_13: Winter Squash [Vegetables, legumes, and soy foods]	-0.092 (0.031), 0.004	-0.058 (0.023), 0.016	-0.028 (0.027), 0.300
C5S_FF_13: Yams/Sweet Potatoes [Vegetables, legumes, and soy foods]	-0.102 (0.035), 0.005	-0.071 (0.034), 0.040	-0.045 (0.031), 0.151
C5U_FF_13: Spinach Raw [Vegetables, legumes, and soy foods]	-0.109 (0.045), 0.019	-0.054 (0.032), 0.099	-0.019 (0.025), 0.456
C5V_FF_13: Kale/Mustard/Chard Greens [Vegetables, legumes, and soy foods]	-0.113 (0.035), 0.002	-0.081 (0.034), 0.021	-0.048 (0.029), 0.104
C5W_FF_13: Head Lettuce [Vegetables, legumes, and soy foods]	-0.076 (0.031), 0.018	-0.062 (0.025), 0.019	-0.038 (0.022), 0.089
C5X_FF_13: Leaf Lettuce [Vegetables, legumes, and soy foods]	-0.220 (0.037), <0.001	-0.127 (0.030), <0.001	-0.065 (0.024), 0.010
C5Y_FF_13: Celery [Vegetables, legumes, and soy foods]	-0.074 (0.035), 0.040	-0.066 (0.032), 0.044	-0.032 (0.032), 0.329
C5Z_FF_13: Peppers [Vegetables, legumes, and soy foods]	-0.154 (0.030), <0.001	-0.121 (0.025), <0.001	-0.061 (0.022), 0.008
Food-group variables: Eggs, meats, and seafood/protein foods			
C6G_FF_13: Chicken/Turkey Wo Skin [Eggs, meats, and seafood/protein foods]	-0.073 (0.026), 0.007	-0.025 (0.029), 0.399	-0.003 (0.024), 0.919
C6I_FF_13: Chicken/Turkey Hot Dogs [Eggs, meats, and seafood/protein foods]	0.069 (0.030), 0.024	0.026 (0.029), 0.375	-0.001 (0.027), 0.983
C6K_FF_13: Processed Meat Other [Eggs, meats, and seafood/protein foods]	0.122 (0.034), 0.001	0.067 (0.031), 0.036	0.023 (0.033), 0.490
C6L_FF_13: Hamburger Lean [Eggs, meats, and seafood/protein foods]	0.068 (0.031), 0.035	0.064 (0.025), 0.013	0.017 (0.025), 0.506
C6M_FF_13: Hamburger Regular [Eggs, meats, and seafood/protein foods]	0.083 (0.035), 0.023	0.058 (0.034), 0.094	0.008 (0.038), 0.838
C6R_FF_13: Liver Chicken/Turkey [Eggs, meats, and seafood/protein foods]	0.127 (0.035), 0.001	0.072 (0.031), 0.024	0.034 (0.024), 0.167
C6U_FF_13: Seafood Main Dish [Eggs, meats, and seafood/protein foods]	-0.118 (0.043), 0.008	-0.053 (0.033), 0.116	-0.036 (0.026), 0.178
C6V_FF_13: Fish Dark [Eggs, meats, and seafood/protein foods]	-0.214 (0.036), <0.001	-0.143 (0.033), <0.001	-0.081 (0.030), 0.010
C6W_FF_13: Fish Other [Eggs, meats, and seafood/protein foods]	-0.170 (0.034), <0.001	-0.113 (0.027), <0.001	-0.079 (0.022), 0.001
Food-group variables: Grains, breads, starches, and savory snacks			
C7E_FF_13: White Bread [Grains, breads, starches, and savory snacks]	0.110 (0.043), 0.013	0.040 (0.034), 0.238	0.018 (0.036), 0.622
C7I_FF_13: Muffins/Biscuits [Grains, breads, starches, and savory snacks]	0.107 (0.041), 0.012	0.087 (0.026), 0.002	0.071 (0.034), 0.039
C7J_FF_13: Rice Brown [Grains, breads, starches, and savory snacks]	-0.125 (0.032), <0.001	-0.085 (0.024), 0.001	-0.053 (0.022), 0.019
C7K_FF_13: Rice White [Grains, breads, starches, and savory snacks]	0.080 (0.033), 0.017	0.064 (0.026), 0.016	0.042 (0.025), 0.096
C7L_FF_13: Pasta [Grains, breads, starches, and savory snacks]	0.079 (0.033), 0.020	0.077 (0.029), 0.011	0.055 (0.032), 0.093
C7N_FF_13: Grains Other [Grains, breads, starches, and savory snacks]	-0.074 (0.036), 0.046	-0.037 (0.025), 0.147	-0.028 (0.027), 0.307
C7Q_FF_13: Potatoes [Grains, breads, starches, and savory snacks]	0.073 (0.036), 0.048	0.077 (0.031), 0.017	0.069 (0.032), 0.036
C7SA_FF_13: Crackers Whole Grain [Grains, breads, starches, and savory snacks]	-0.104 (0.037), 0.007	-0.050 (0.036), 0.164	-0.045 (0.030), 0.148
C7T_FF_13: Pizza [Grains, breads, starches, and savory snacks]	0.099 (0.034), 0.005	0.086 (0.032), 0.010	0.064 (0.033), 0.056
Food-group variables: Beverages and alcohol			
C8A_FF_13: Low Cal Carbonated W Caffeine [Beverages and alcohol]	0.076 (0.033), 0.023	0.077 (0.028), 0.009	0.019 (0.022), 0.395
C8B_FF_13: Low Cal Carbonated Wo Caffeine [Beverages and alcohol]	0.082 (0.028), 0.005	0.083 (0.030), 0.007	0.037 (0.028), 0.190
C8C_FF_13: Carbonated W Caffeine And Sugar [Beverages and alcohol]	0.132 (0.029), <0.001	0.098 (0.031), 0.003	0.112 (0.034), 0.002
C8D_FF_13: Carbonated W Sugar Other [Beverages and alcohol]	0.064 (0.030), 0.037	0.039 (0.028), 0.162	0.037 (0.026), 0.165
C8E_FF_13: Sugar Beverage Other [Beverages and alcohol]	0.079 (0.039), 0.050	0.059 (0.036), 0.110	0.038 (0.026), 0.150
C8H_FF_13: Red Wine [Beverages and alcohol]	-0.128 (0.024), <0.001	-0.053 (0.025), 0.044	-0.017 (0.029), 0.569
C8I_FF_13: White Wine [Beverages and alcohol]	-0.130 (0.026), <0.001	-0.058 (0.020), 0.006	-0.021 (0.020), 0.301
C8K_FF_13: Plain Water [Beverages and alcohol]	-0.097 (0.038), 0.013	-0.092 (0.031), 0.005	-0.050 (0.028), 0.073
C8N_FF_13: Decaf Coffee [Beverages and alcohol]	-0.067 (0.031), 0.034	-0.102 (0.026), <0.001	-0.086 (0.026), 0.001
Food-group variables: Sweets, snacks, condiments, and added fats			
C9B_FF_13: Dark Chocolate [Sweets, snacks, condiments, and added fats]	-0.092 (0.029), 0.002	-0.077 (0.023), 0.002	-0.042 (0.021), 0.044
C9D_FF_13: Candy Wo Chocolate [Sweets, snacks, condiments, and added fats]	0.104 (0.026), <0.001	0.087 (0.019), <0.001	0.077 (0.028), 0.008
C9N_FF_13: Doughnuts [Sweets, snacks, condiments, and added fats]	0.087 (0.037), 0.024	0.063 (0.035), 0.075	0.047 (0.033), 0.151
C9O_FF_13: Cake Home Baked [Sweets, snacks, condiments, and added fats]	0.067 (0.028), 0.022	0.068 (0.025), 0.009	0.057 (0.026), 0.034
C9T_FF_13: Pastry Store [Sweets, snacks, condiments, and added fats]	0.139 (0.047), 0.004	0.101 (0.039), 0.012	0.082 (0.029), 0.006
C9V_FF_13: Peanuts [Sweets, snacks, condiments, and added fats]	-0.112 (0.028), <0.001	-0.088 (0.025), 0.001	-0.064 (0.020), 0.002
C9W_FF_13: Walnuts [Sweets, snacks, condiments, and added fats]	-0.174 (0.029), <0.001	-0.143 (0.027), <0.001	-0.092 (0.024), <0.001
C9X_FF_13: Nuts Other [Sweets, snacks, condiments, and added fats]	-0.197 (0.026), <0.001	-0.137 (0.025), <0.001	-0.090 (0.027), 0.002
C9AB_FF_13: Oat Bran Added [Sweets, snacks, condiments, and added fats]	-0.085 (0.026), 0.001	-0.071 (0.016), <0.001	-0.059 (0.017), 0.001
C9AG_FF_13: Salt Added [Sweets, snacks, condiments, and added fats]	0.072 (0.033), 0.033	0.074 (0.031), 0.020	0.047 (0.026), 0.082
C9AH_FF_13: Number Tsps Sugar [Sweets, snacks, condiments, and added fats]	0.091 (0.037), 0.017	0.005 (0.035), 0.895	0.002 (0.030), 0.936
C9AL_FF_13: Mayonnaise Fat Free [Sweets, snacks, condiments, and added fats]	0.058 (0.044), 0.191	0.054 (0.039), 0.170	0.022 (0.033), 0.505
C9AM_FF_13: Mayonnaise Regular [Sweets, snacks, condiments, and added fats]	0.077 (0.036), 0.037	0.051 (0.030), 0.090	0.055 (0.031), 0.080
C9AN_FF_13: Salad Dressing [Sweets, snacks, condiments, and added fats]	-0.086 (0.036), 0.020	-0.008 (0.034), 0.807	0.018 (0.029), 0.535

Values are β coefficients (standard errors) from survey-weighted linear regression models with frailty score (fried_sum; range 0–5) as the outcome. All dietary predictors were standardized as z-scores; thus, β estimates represent the mean difference in frailty score per 1-standard deviation higher dietary exposure. All continuous dietary predictors were standardized within the final analytic sample as z-scores; β coefficients therefore represent the mean difference in frailty score associated with a 1-SD higher dietary value. Negative β values indicate lower frailty burden. Model 1 adjusted for age and sex. Model 2 additionally adjusted for race/ethnicity, marital status, education, and household income. Model 3 further adjusted for smoking status, alcohol use, physical activity, body mass index, hypertension, diabetes, stroke, heart disease, and self-rated health. Analyses used the HRS complex survey design with HCNSWGTR_NT sampling weights, stratum as the stratification variable, secu as the primary sampling unit, and single-unit strata centered at the overall mean. Dietary variables were derived from the 2013 Health Care and Nutrition Study food frequency questionnaire. HEI-2015 component labels and food-group labels were mapped from the uploaded dietary codebook. HEI-2015 component scores reflect greater alignment with the Dietary Guidelines for Americans and include adequacy components (total vegetables, greens and beans, total fruit, whole fruit, whole grains, dairy, total protein foods, seafood and plant proteins, and fatty acid ratio) and moderation components (sodium, refined grains, added sugar, and saturated fat). Food-group codes C3–C9 correspond to the major domains defined in the codebook. P-values are two-sided and shown as <0.001 when appropriate

**Fig. 2:**
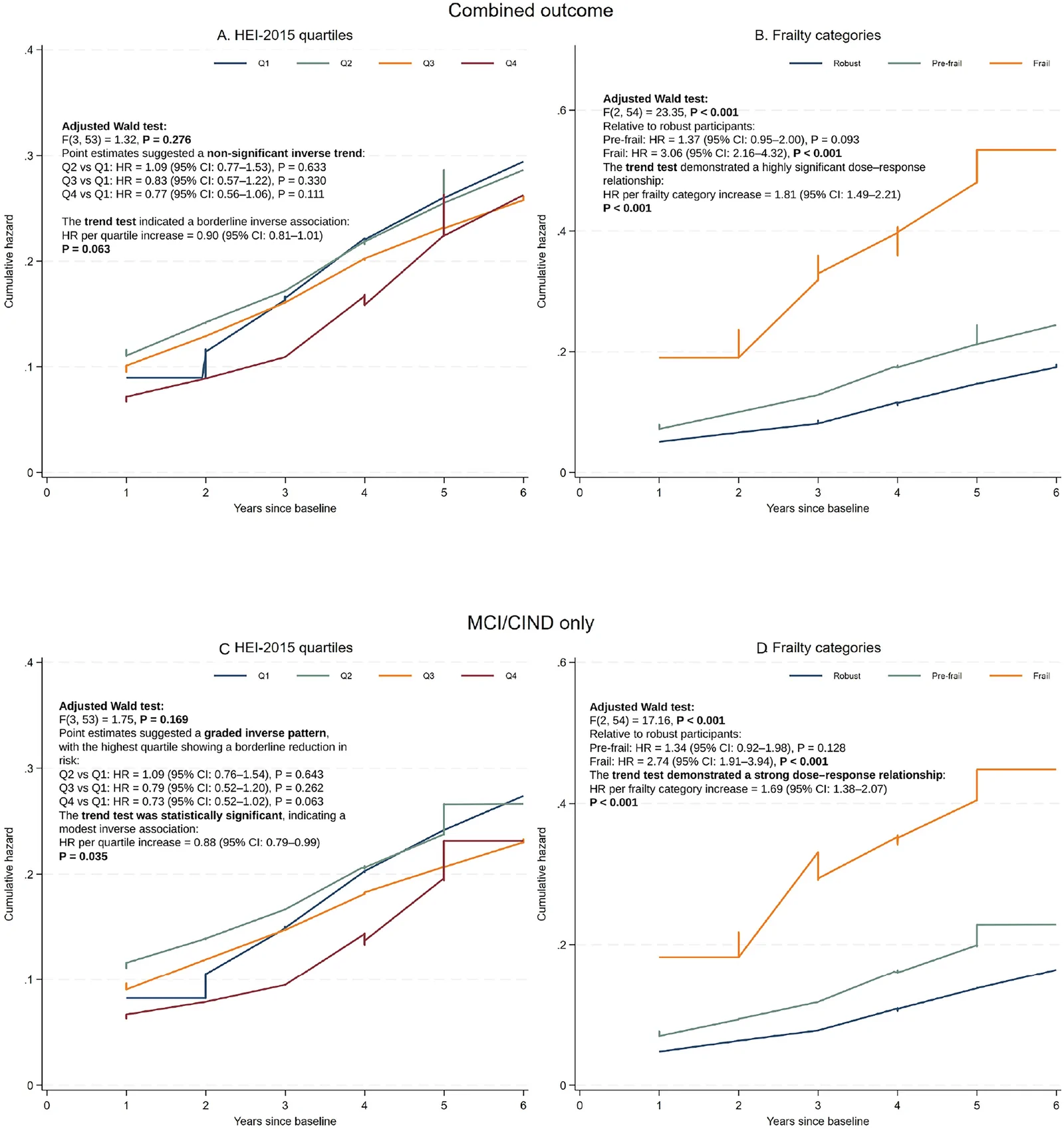
Associations of diet quality and frailty with incident cognitive impairment. Survey-weighted Cox associations are shown for **(A)** HEI-2015 quartiles and composite CIND/probable dementia, **(B)** frailty status and composite CIND/probable dementia, **(C)** HEI-2015 quartiles and MCI/CIND, and **(D)** frailty status and MCI/CIND. Q1 and robust status were selected as the lowest ordered categories of their respective measures, allowing comparisons with progressively higher diet quality and greater frailty, respectively. HEI-2015 quartiles were Q1, ≤63.80; Q2, >63.80–70.25; Q3, >70.25–76.70; and Q4, >76.70 (reference: Q1). Frailty was classified as robust (0 components), pre-frail (1–2), or frail ( ≥ 3; reference: robust). For composite CIND/dementia, HEI-2015 was not associated with risk (global P = 0.276; Q4 vs Q1: HR = 0.77, 95% CI: 0.56–1.06; trend P = 0.063). Frailty was strongly associated with risk (global P < 0.001; frail vs robust: HR = 3.06, 95% CI: 2.16–4.32; trend HR = 1.81, P < 0.001). For MCI/CIND alone, HEI-2015 showed an inverse trend (HR per quartile=0.88, 95% CI: 0.79–0.99; P = 0.035), while frailty remained strongly associated (frail vs robust: HR = 2.74, 95% CI: 1.91–3.94; trend HR = 1.69, P < 0.001). CI = confidence interval; CIND = cognitively impaired non-dementia; HEI-2015 = Healthy Eating Index–2015; HR = hazard ratio

Figure 2 illustrates associations of diet quality (HEI-2015) and frailty with incident cognitive impairment. HEI-2015 was not significantly associated with the composite CIND/probable dementia outcome (Fig. 2A; global P = 0.276), whereas frailty showed a strong graded association with this outcome (Fig. 2B; frail vs robust: Hazard Ratio (HR) = 3.06, 95% confidence interval (CI): 2.16–4.32; trend HR = 1.81, P < 0.001). For MCI/CIND, increasing HEI-2015 quartile was associated with lower risk (Fig. 2C; HR per one-category increase=0.88, 95% CI: 0.79–0.99; P for trend=0.035), while frailty remained strongly associated with higher risk (Fig. 2D; frail vs robust: HR = 2.74, 95% CI: 1.91–3.94; trend HR = 1.69, P < 0.001).

Table 3 presents survey-weighted Cox proportional hazards models examining the association between frailty status and incident cognitive outcomes, including composite (CIND/probable dementia), MCI/CIND-only, and probable dementia-only. In age- and sex-adjusted models (Model 1), frailty (frail vs. robust) was strongly associated with higher risk of all outcomes, particularly for the composite outcome (HR = 2.27, 95% CI: 1.56–3.32, p < 0.001) and MCI/CIND (HR = 2.15, 95% CI: 1.45–3.17, p < 0.001), with elevated risk for probable dementia (HR = 5.66, 95% CI: 1.14–28.12, p = 0.035). These associations were progressively attenuated with further adjustment. In Model 2, frailty (frail vs. robust) remained associated with the composite outcome and MCI/CIND, though effect sizes were reduced, while the dementia estimate remained imprecise. In the fully adjusted model (Model 3), associations were further attenuated and no longer statistically significant upon adjustment for other lifestyle and health-related factors (See Covariates section for details). Pre-frailty was not associated with any cognitive outcome across models.

**Table 3:** Frailty and incident cognitive outcomes (HRS, n = 1,636)

Outcome	Exposure	Model 1 HR (95% CI), p	Model 2 HR (95% CI), p	Model 3 HR (95% CI), p
CIND/Probable dementia	Pre-frail vs Robust	1.281 (0.873–1.880), 0.202	1.096 (0.726–1.653), 0.658	0.995 (0.661–1.497), 0.980
CIND/Probable dementia	Frail vs Robust	2.272 (1.556–3.316), <0.001	1.685 (1.162–2.443), 0.007	1.365 (0.893–2.085), 0.148
MCI/CIND only	Pre-frail vs Robust	1.273 (0.859–1.886), 0.224	1.072 (0.704–1.632), 0.742	0.964 (0.632–1.471), 0.864
MCI/CIND only	Frail vs Robust	2.145 (1.452–3.169), <0.001	1.514 (1.027–2.230), 0.037	1.190 (0.765–1.849), 0.434
Probable dementia only	Pre-frail vs Robust	1.731 (0.441–6.791), 0.425	1.677 (0.396–7.101), 0.476	1.285 (0.309–5.345), 0.726
Probable dementia only	Frail vs Robust	5.661 (1.140–28.119), 0.035	6.003 (1.337–26.958), 0.020	3.836 (0.761–19.346), 0.102

Hazard ratios (HR) and 95% confidence intervals from survey-weighted Cox models. Model 1 adjusted for age and sex. Model 2 additionally adjusted for race/ethnicity, education, marital status, and income. Model 3 further adjusted for lifestyle factors, chronic conditions, and HEI-2015. Analyses accounted for HRS complex survey design

**Fig. 3:**
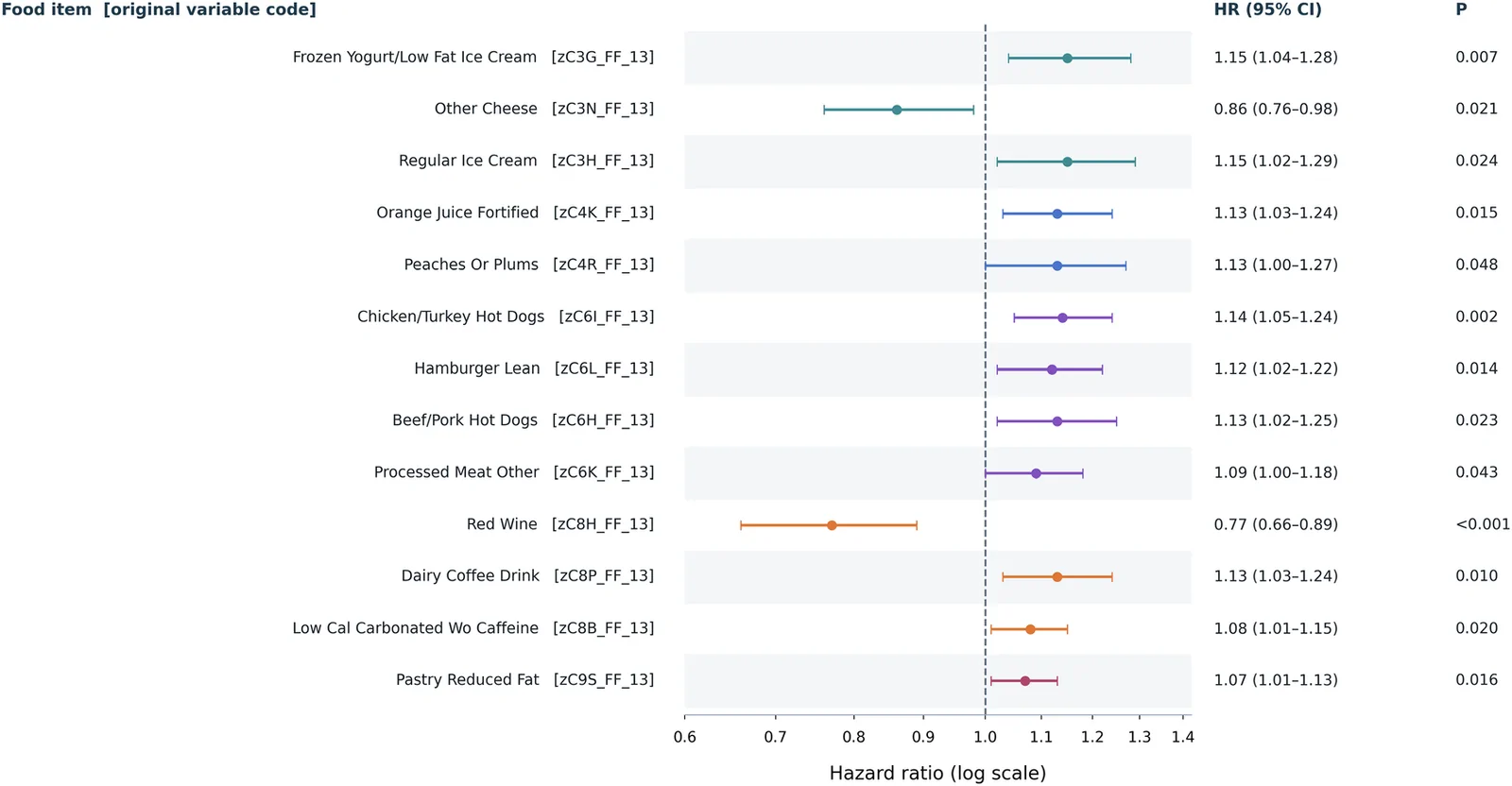
Associations of selected individual dietary food items with incident cognitive impairment in the Health and Retirement Study: Fully adjusted Model 3. Forest plot showing adjusted hazard ratios (HRs) and 95% confidence intervals (CIs) for associations between individual dietary food items and incident cognitive impairment, defined as the first occurrence of cognitively impaired non-dementia (CIND) or probable dementia during follow-up through 2022. The figure presents 13 food items with nominally significant associations (P < 0.05) in the fully adjusted Model 3, grouped according to their original dietary categories: C3, dairy, dairy alternatives, and spreads/fats; C4, fruits and fruit juices; C6, eggs, meats, seafood, and other protein foods; C8, beverages and alcohol; and C9, sweets, snacks, condiments, and added fats. Dietary intake was assessed using the 2013 Health Care and Nutrition Study food-frequency questionnaire. All dietary exposures were standardized, and HRs represent associations per 1-standard-deviation higher intake. Model 3 adjusted for age, sex, race/ethnicity, marital status, education, household income, smoking status, alcohol use, physical activity, body mass index, hypertension, diabetes, stroke, heart disease, and self-rated health. Analyses used survey-weighted Cox proportional hazards models accounting for the complex HRS sampling design. Points represent adjusted HRs, horizontal bars indicate 95% CIs, and the dashed vertical line denotes HR = 1.0. HRs below 1.0 indicate lower hazard, whereas HRs above 1.0 indicate higher hazard. Although all displayed associations met nominal statistical significance (P < 0.05), none remained significant after Benjamini–Hochberg false discovery rate (FDR) correction (q < 0.05). Accordingly, these findings should be interpreted as exploratory rather than confirmatory. Complete results, including FDR-adjusted q-values, are provided in Supplementary Datasheet 3, and dietary variable definitions are provided in Supplementary Datasheet 1. CI, confidence interval; CIND, cognitively impaired non-dementia; FDR, false discovery rate; HR, hazard ratio; HRS, Health and Retirement Study

Secondary exploratory analyses of individual HEI-2015 components and food items revealed heterogeneous associations with incident cognitive impairment (Fig. 3). In fully adjusted Cox models (Model 3), 13 individual food items across five dietary groups were nominally associated (P < 0.05) with the composite outcome of incident CIND/probable dementia. Within dairy products (C3), frozen yogurt/low-fat ice cream (HR = 1.15, 95% CI: 1.04–1.28; P = 0.007) and regular ice cream (HR = 1.15, 95% CI: 1.02–1.29; P = 0.024) were associated with higher risk, whereas other cheese was inversely associated (HR = 0.86, 95% CI: 0.76–0.98; P = 0.021). Among fruits and juices (C4), fortified orange juice (HR = 1.13, 95% CI: 1.03–1.24; P = 0.015) and peaches/plums (HR = 1.13, 95% CI: 1.00–1.27; P = 0.048) showed positive associations. Within meat and protein foods (C6), chicken/turkey hot dogs (HR = 1.14, 95% CI: 1.05–1.24; P = 0.002), lean hamburgers (HR = 1.12, 95% CI: 1.02–1.22; P = 0.014), beef/pork hot dogs (HR = 1.13, 95% CI: 1.02–1.25; P = 0.023), and other processed meats (HR = 1.09, 95% CI: 1.00–1.18; P = 0.043) were associated with higher risk. Among beverages (C8), red wine exhibited the strongest inverse association (HR = 0.77, 95% CI: 0.66–0.89; P < 0.001), whereas dairy coffee drinks (HR = 1.13, 95% CI: 1.03–1.24; P = 0.010) and low-calorie caffeine-free carbonated beverages (HR = 1.08, 95% CI: 1.01–1.15; P = 0.020) showed positive associations. Reduced-fat pastries (C9) were also positively associated (HR = 1.07, 95% CI: 1.01–1.13; P = 0.016).

Despite these nominal associations, none remained statistically significant after Benjamini–Hochberg FDR correction for the composite CIND/probable dementia outcome or MCI/CIND alone. For probable dementia alone, five associations survived FDR correction (q < 0.05): the HEI-2015 added-sugar component score, raisins/grapes (C4A; HR = 1.53, 95% CI: 1.30–1.80), cooked carrots (C5L; HR = 0.49, 95% CI: 0.33–0.73), chicken/turkey hot dogs (C6I; HR = 1.28, 95% CI: 1.13–1.45), and dairy coffee drinks (C8P; HR = 1.31, 95% CI: 1.12–1.54). However, the added-sugar estimate was unstable and uninterpretable, and the remaining associations should be considered exploratory given the limited number of probable dementia events and potential residual confounding. Complete nominal and FDR-adjusted results are provided in Supplementary Materials 3: Supplementary Datasheet 3 and Supplementary Materials 2**:** Figure S1, with dietary item definitions in Supplementary Materials 3: Supplementary Datasheet 1.

**Fig. 4:**
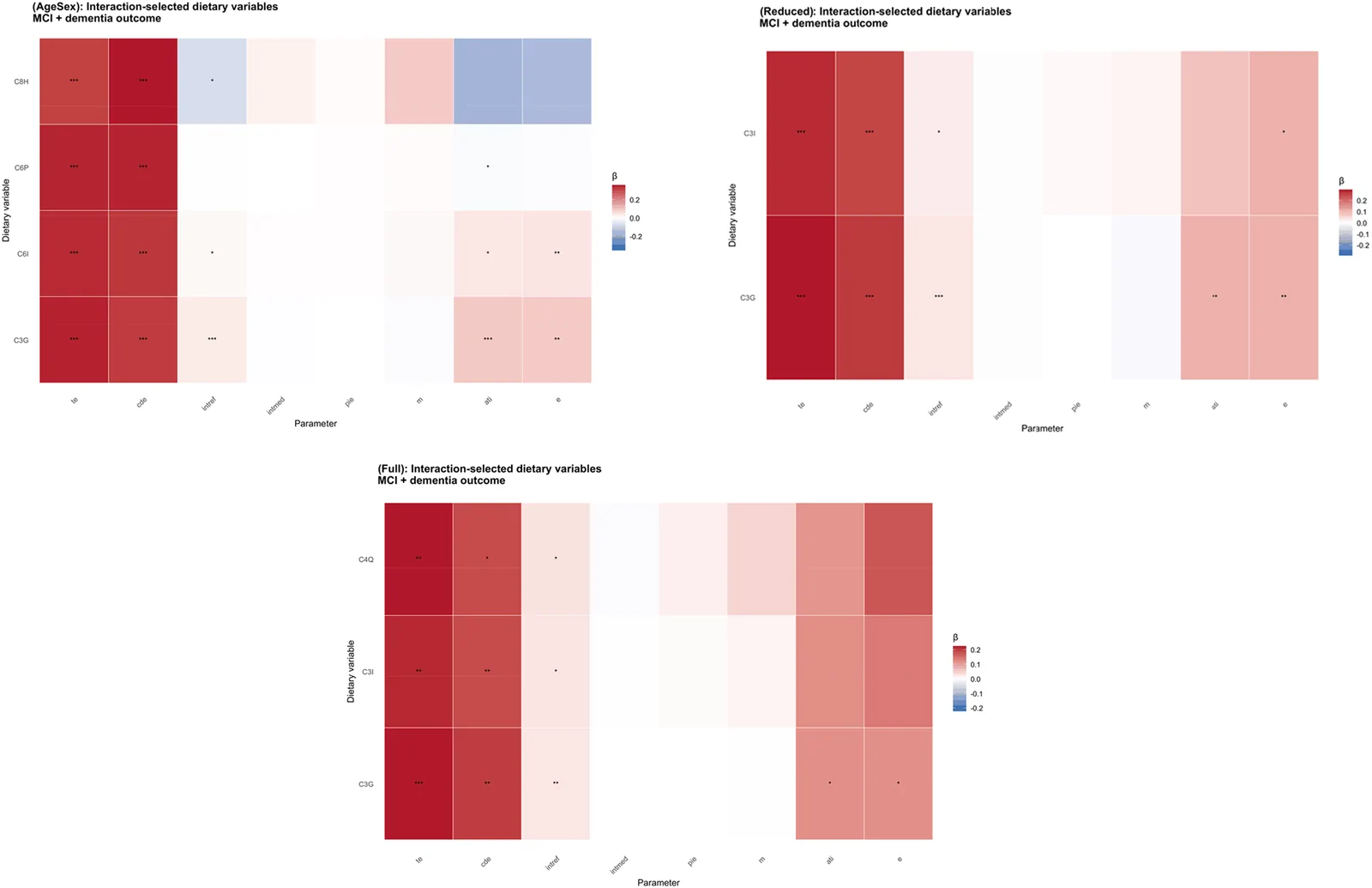
Heatmap of four-way decomposition results highlighting interaction components and food groups. Heatmap of selected four-way decompositions examining frailty as the exposure and diet as the mediator and effect modifier for incident CIND/dementia in HRS, 2016–2022. Rows represent HEI-2015 and selected C3–C9 food groups with at least one significant interaction component; columns show estimates from med4way using survey-weighted Cox outcome and linear mediator models. Parameters include the controlled direct effect (CDE), pure indirect effect (PIE), reference interaction (INTref), mediated interaction (INTmed), total effect (TE), attributable proportion due to interaction (ATI), and related proportions. Red and blue indicate higher and lower risk, respectively; asterisks denote statistical significance. Positive and negative interaction estimates suggest synergism and antagonism, respectively. All food groups are listed in Supplementary Datasheet 1. Because diet was assessed before frailty, decomposition components involving mediation are presented descriptively and are not interpreted as evidence of causal mediation

Figure 4 and S2 jointly summarize the four-way decomposition analyses of frailty and diet (HEI-2015 and food-group variables) in relation to incident cognitive impairment. Within these exploratory decompositions, the controlled direct effect (CDE) was generally the largest component, whereas the pure indirect effect (PIE) and mediated interaction (INTmed) components were generally small. For the HEI-2015 total score, no statistically significant mediation or interaction components were detected across models. For specific food groups and the composite outcome (CIND/probable dementia), statistically significant reference interaction (INTref) components in the fully adjusted model were limited to three items: frozen yogurt/low-fat ice cream (C3G; β = 0.026, *P* = 0.007), flavored yogurt (C3I; β = 0.027, *P* = 0.015), and other berries (C4Q; β = 0.029, *P* = 0.049). In age- and sex-adjusted models, additional INTref signals were observed for chicken/turkey hot dogs (C6I; *P* = 0.039) and red wine (C8H; *P* = 0.048). Red wine also showed a significant INTref component in the fully adjusted MCI/CIND-only model (*P* = 0.041). Mediation signals (Supplementary Materials 2: Figure S2**)** were limited to a small number of food items. For the composite outcome, PIE components were statistically significant for other berries (C4Q) and raw carrots (C5K) in the age- and sex-adjusted model and remained significant in the reduced model for both items; neither remained significant after full adjustment. For the probable dementia–only outcome, INTref components were statistically significant across all three adjustment levels for salsa (C5D), tofu/soy protein (C5E), beef/pork/lamb mixed dishes (C6N), and three snack/condiment items (C7N, C9AA, and C9AD). No PIE component remained statistically significant after full adjustment for this outcome. Processed meat variables such as C6J showed neither statistically significant mediation nor interaction components. Overall, the CDE associated with frailty was generally the predominant component across outcomes and adjustment levels, whereas dietary factors showed relatively few and heterogeneous interaction or indirect-effect signals. Given the temporal ordering of diet and frailty, these findings are interpreted as exploratory decompositions of association rather than evidence of causal mediation. The full set of results is presented in Supplementary Materials 3: Supplementary Datasheet 4.

Across these analyses, higher diet quality was consistently associated with lower frailty, and this association persisted after adjustment for sociodemographic, behavioral, and clinical factors. In contrast, frailty was strongly associated with incident cognitive impairment in minimally adjusted models, but the estimates attenuated and were no longer statistically significant after full adjustment. Overall HEI-2015 was not associated with incident cognitive impairment, although selected food groups showed heterogeneous associations. Exploratory four-way decomposition analyses, conducted only with frailty as the exposure and diet as the mediator/effect modifier, provided little evidence of indirect effects through diet, while interaction between frailty and dietary factors was limited and heterogeneous. Given that diet was assessed before frailty, these decompositions are interpreted as exploratory analyses of association and effect modification rather than causal mediation.

**Fig. 5:**
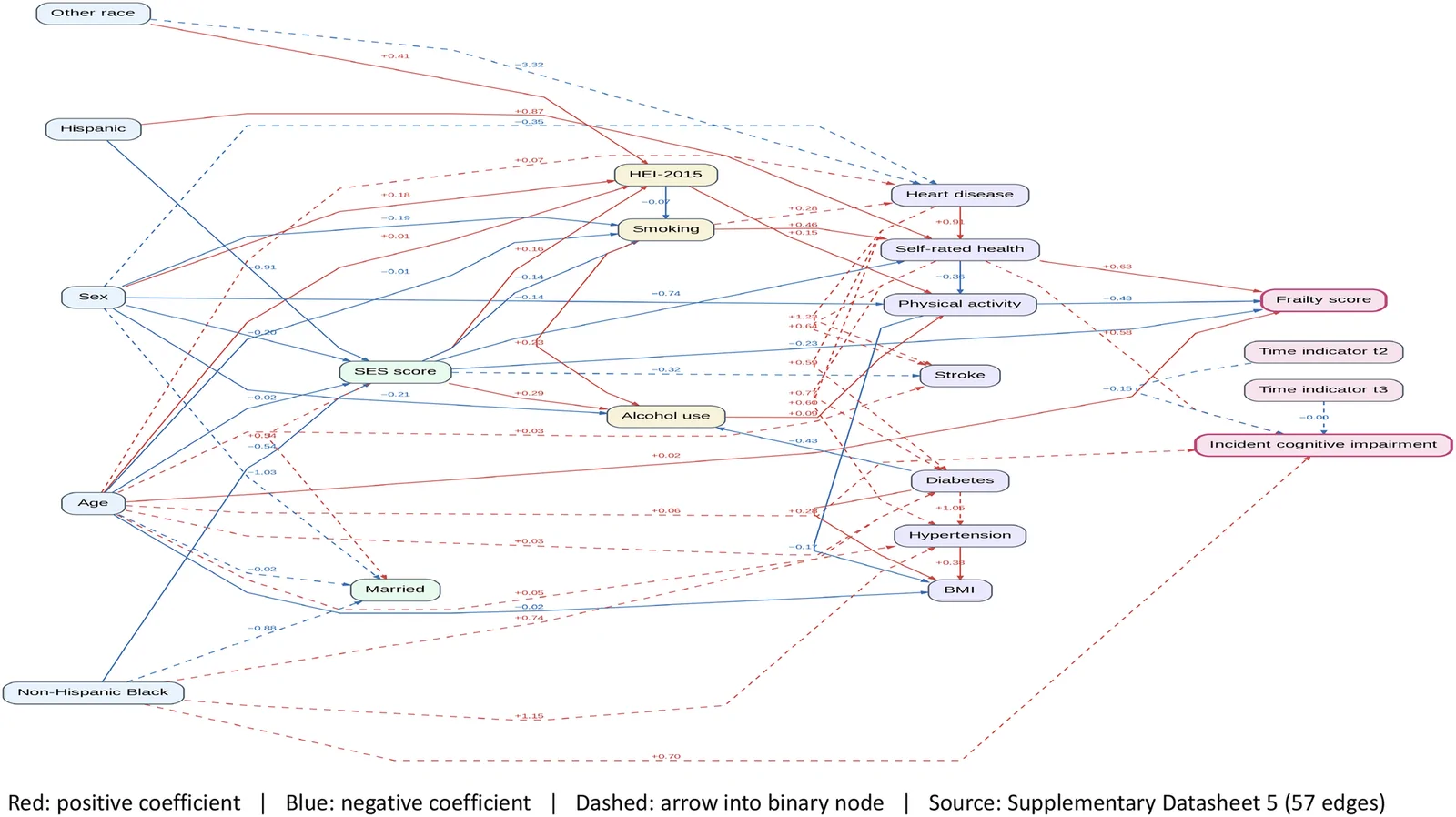
Additive Bayesian network of diet quality, frailty, and incident cognitive impairment. The additive Bayesian network (ABN) illustrates 57 directed conditional dependencies among demographic, socioeconomic, dietary, behavioral, and clinical factors, frailty, and incident cognitive impairment in the Health and Retirement Study. Dietary quality was assessed using the Healthy Eating Index–2015 (HEI-2015) in 2013, frailty was assessed in 2016, and incident cognitive impairment (CIND/probable dementia) was evaluated through 2022. Nodes are grouped by variable domain, and directed arrows represent the relationships identified by the ABN. Numerical labels indicate node-specific regression coefficients, with red and blue arrows representing positive and negative associations, respectively; dashed arrows identify relationships directed toward binary outcome nodes. HEI-2015 was positioned upstream of behavioral and health-related factors, while frailty was connected to socioeconomic status, age, physical activity, self-rated health, and other clinical characteristics. The direct parent nodes of incident cognitive impairment were age, non-Hispanic Black race, self-rated health, and follow-up indicators (t2 and t3). Neither HEI-2015 nor frailty had a direct edge to incident cognitive impairment in the full ABN. These conditional dependencies describe the network structure and should not be interpreted as evidence of causal relationships. Complete edge coefficients and variable definitions are provided in Supplementary Materials 3: Supplementary Datasheet 5. ABN, additive Bayesian network; BMI, body mass index; CIND, cognitively impaired non-dementia; HEI-2015, Healthy Eating Index–2015; HRS, Health and Retirement Study; SES, socioeconomic status

Figure 5 and Supplementary Materials 3: supplementary datasheet 5 depict the ABN structure for the CIND/probable dementia outcome with HEI-2015 total score as the main dietary exposure and frailty as an intermediate network node. Frailty occupied a central upstream position linking physical activity, BMI, self-rated health, socioeconomic status, and chronic conditions. However, in the ABN-informed GSEM, frailty was not retained as direct parent of cognitive survival node after omission of time indicators. Instead, direct parents of the survival node were age, non-Hispanic Black race, and self-rated health. HEI-2015 appeared upstream and was not directly connected to incident cognitive impairment. These findings support a model in which diet and frailty are embedded within broader sociodemographic, behavioral, and health-related pathways, rather than providing evidence that frailty directly predicts cognitive impairment in the final ABN-informed GSEM.

GSEM models informed by the additive Bayesian network, presented in Table 4, indicated that frailty occupied a central position linking socioeconomic, behavioral, and clinical factors to incident cognitive impairment. Direct predictors of the survival outcome included age, non-Hispanic Black race, and self-rated health, while frailty showed strong upstream connections with physical activity, body mass index, and chronic conditions. HEI-2015 was associated with socioeconomic and behavioral variables but was not directly linked to cognitive impairment. Overall, Table 4 supports an upstream role for frailty within the network, but not a direct effect of frailty on the cognitive survival outcome in the final ABN-informed GSEM.

**Table 4:** Additive Bayesian network–informed generalized structural equation model for incident cognitive impairment (MCI/CIND or dementia) in the health and retirement study

Response node	Predictor	Family	Link	Effect type	Estimate	95% CI lower	95% CI upper	P value	Raw coefficient	SE	z
Incident cognitive impairment (MCI/CIND or dementia)	Age (years)	Weibull	Log	HR	1.078	1.066	1.092	0.000	0.076	0.006	12.280
Incident cognitive impairment (MCI/CIND or dementia)	Non-Hispanic Black	Weibull	Log	HR	2.004	1.522	2.639	0.000	0.695	0.140	4.950
Incident cognitive impairment (MCI/CIND or dementia)	Self-rated health, 2012	Weibull	Log	HR	2.152	1.687	2.746	0.000	0.767	0.124	6.170
SES factor score, 2012	Age (years)	Gaussian	Identity	Beta	-0.016	-0.021	-0.011	0.000	-0.016	0.003	-6.350
SES factor score, 2012	Sex	Gaussian	Identity	Beta	-0.215	-0.309	-0.121	0.000	-0.215	0.048	-4.480
SES factor score, 2012	Non-Hispanic Black	Gaussian	Identity	Beta	-0.563	-0.703	-0.423	0.000	-0.563	0.071	-7.910
SES factor score, 2012	Hispanic	Gaussian	Identity	Beta	-0.884	-1.048	-0.719	0.000	-0.884	0.084	-10.530
Married/partnered, 2012	Age (years)	Bernoulli	Logit	OR	0.985	0.973	0.998	0.019	-0.015	0.006	-2.350
Married/partnered, 2012	Sex	Bernoulli	Logit	OR	0.350	0.271	0.452	0.000	-1.050	0.130	-8.070
Married/partnered, 2012	Non-Hispanic Black	Bernoulli	Logit	OR	0.406	0.295	0.558	0.000	-0.901	0.163	-5.540
Married/partnered, 2012	SES factor score, 2012	Bernoulli	Logit	OR	1.733	1.531	1.961	0.000	0.550	0.063	8.720
HEI-2015 total score (continuous z-score)	SES factor score, 2012	Gaussian	Identity	Beta	0.169	0.121	0.217	0.000	0.169	0.024	6.890
HEI-2015 total score (continuous z-score)	Age (years)	Gaussian	Identity	Beta	0.008	0.003	0.013	0.003	0.008	0.003	2.920
HEI-2015 total score (continuous z-score)	Sex	Gaussian	Identity	Beta	0.206	0.108	0.304	0.000	0.206	0.050	4.120
HEI-2015 total score (continuous z-score)	Other race/ethnicity	Gaussian	Identity	Beta	0.426	0.157	0.694	0.002	0.426	0.137	3.100
Smoking status, 2012	Age (years)	Ordinal	Logit	OR	0.981	0.971	0.991	0.000	-0.019	0.005	-3.600
Smoking status, 2012	Sex	Ordinal	Logit	OR	0.531	0.437	0.647	0.000	-0.632	0.100	-6.320
Smoking status, 2012	SES factor score, 2012	Ordinal	Logit	OR	0.639	0.577	0.708	0.000	-0.447	0.052	-8.630
Smoking status, 2012	HEI-2015 total score (continuous z-score)	Ordinal	Logit	OR	0.834	0.757	0.919	0.000	-0.181	0.049	-3.680
Alcohol use, 2012	Sex	Ordinal	Logit	OR	0.640	0.530	0.774	0.000	-0.446	0.097	-4.610
Alcohol use, 2012	SES factor score, 2012	Ordinal	Logit	OR	1.632	1.480	1.800	0.000	0.490	0.050	9.800
Alcohol use, 2012	Smoking status, 2012	Ordinal	Logit	OR	1.496	1.300	1.722	0.000	0.403	0.072	5.610
Alcohol use, 2012	Diabetes, 2012	Ordinal	Logit	OR	0.478	0.373	0.614	0.000	-0.737	0.127	-5.800
Self-rated health, 2012	Hispanic	Ordinal	Logit	OR	2.293	1.538	3.419	0.000	0.830	0.204	4.070
Self-rated health, 2012	SES factor score, 2012	Ordinal	Logit	OR	0.499	0.429	0.580	0.000	-0.695	0.077	-9.080
Self-rated health, 2012	Smoking status, 2012	Ordinal	Logit	OR	1.494	1.227	1.819	0.000	0.401	0.100	4.000
Self-rated health, 2012	Heart disease, 2012	Ordinal	Logit	OR	2.292	1.693	3.103	0.000	0.829	0.155	5.370
Physical activity, 2012	Sex	Ordinal	Logit	OR	0.680	0.552	0.837	0.000	-0.386	0.106	-3.640
Physical activity, 2012	Alcohol use, 2012	Ordinal	Logit	OR	1.314	1.198	1.441	0.000	0.273	0.047	5.810
Physical activity, 2012	Self-rated health, 2012	Ordinal	Logit	OR	0.392	0.306	0.502	0.000	-0.937	0.127	-7.400
Physical activity, 2012	HEI-2015 total score (continuous z-score)	Ordinal	Logit	OR	1.546	1.397	1.711	0.000	0.436	0.052	8.440
Diabetes, 2012	Age (years)	Bernoulli	Logit	OR	1.020	1.006	1.035	0.006	0.020	0.007	2.750
Diabetes, 2012	Non-Hispanic Black	Bernoulli	Logit	OR	1.939	1.367	2.749	0.000	0.662	0.178	3.710
Diabetes, 2012	Heart disease, 2012	Bernoulli	Logit	OR	1.711	1.259	2.324	0.001	0.537	0.156	3.440
Diabetes, 2012	Self-rated health, 2012	Bernoulli	Logit	OR	1.939	1.427	2.633	0.000	0.662	0.156	4.240
Heart disease, 2012	Age (years)	Bernoulli	Logit	OR	1.070	1.056	1.085	0.000	0.068	0.007	9.590
Heart disease, 2012	Sex	Bernoulli	Logit	OR	0.670	0.519	0.865	0.002	-0.400	0.130	-3.070
Heart disease, 2012	Other race/ethnicity	Bernoulli	Logit	OR	0.091	0.012	0.666	0.018	-2.401	1.018	-2.360
Heart disease, 2012	Smoking status, 2012	Bernoulli	Logit	OR	1.280	1.053	1.556	0.013	0.247	0.100	2.470
Hypertension, 2012	Age (years)	Bernoulli	Logit	OR	1.045	1.032	1.057	0.000	0.044	0.006	7.330
Hypertension, 2012	Non-Hispanic Black	Bernoulli	Logit	OR	2.979	2.121	4.184	0.000	1.092	0.173	6.300
Hypertension, 2012	Diabetes, 2012	Bernoulli	Logit	OR	2.912	2.150	3.943	0.000	1.069	0.155	6.910
Hypertension, 2012	Heart disease, 2012	Bernoulli	Logit	OR	1.719	1.304	2.267	0.000	0.542	0.141	3.840
BMI category, 2012	Age (years)	Ordinal	Logit	OR	0.958	0.948	0.968	0.000	-0.043	0.005	-8.250
BMI category, 2012	Physical activity, 2012	Ordinal	Logit	OR	0.636	0.556	0.727	0.000	-0.453	0.068	-6.620
BMI category, 2012	Hypertension, 2012	Ordinal	Logit	OR	2.547	2.094	3.098	0.000	0.935	0.100	9.360
BMI category, 2012	Diabetes, 2012	Ordinal	Logit	OR	2.140	1.645	2.785	0.000	0.761	0.134	5.660
Stroke, 2012	Age (years)	Bernoulli	Logit	OR	1.023	0.999	1.047	0.059	0.023	0.012	1.890
Stroke, 2012	SES factor score, 2012	Bernoulli	Logit	OR	0.736	0.585	0.925	0.009	-0.307	0.117	-2.630
Stroke, 2012	Heart disease, 2012	Bernoulli	Logit	OR	3.587	2.273	5.660	0.000	1.277	0.233	5.490
Stroke, 2012	Self-rated health, 2012	Bernoulli	Logit	OR	1.611	0.981	2.645	0.060	0.477	0.253	1.880
Fried frailty sum (continuous)	SES factor score, 2012	Gaussian	Identity	Beta	-0.222	-0.269	-0.175	0.000	-0.222	0.024	-9.250
Fried frailty sum (continuous)	Age (years)	Gaussian	Identity	Beta	0.025	0.020	0.030	0.000	0.025	0.003	9.640
Fried frailty sum (continuous)	Physical activity, 2012	Gaussian	Identity	Beta	-0.437	-0.506	-0.369	0.000	-0.437	0.035	-12.580
Fried frailty sum (continuous)	Self-rated health, 2012	Gaussian	Identity	Beta	0.664	0.534	0.795	0.000	0.664	0.067	9.990

*ABN* additive Bayesian network, *BMI* body mass index, *CIND* cognitively impaired non-dementia, *HEI-2015* healthy eating index–2015, *HRS* health and retirement study, *HR* hazard ratio, *OR* odds ratio, *SES* socioeconomic status, *SRH* self-rated health, *GSEM* generalized structural equation model

Data are from the Health and Retirement Study (HRS), linking 2013 Health Care and Nutrition Study dietary data, 2012 covariates, baseline 2016 cognition and frailty status, and follow-up through 2022. The analytic sample included 1,636 cognitively normal participants at baseline; after survival-time setup (*stset*), 1,515 individuals contributed to the Weibull survival analysis, with 349 incident cognitive impairment events observed over 7,299.08 person-years of follow-up (log likelihood = −19,117.3). The outcome is incident cognitive impairment, defined as the first transition from normal cognition in 2016 to either MCI/CIND or probable dementia during follow-up. The model was additive Bayesian network–informed; after omission of time dummies (t2/t3), direct parents of the survival node were age, non-Hispanic Black race, and self-rated health. Continuous nodes (e.g., socioeconomic status, HEI-2015 total score, and Fried frailty sum) were modeled using Gaussian identity links; Bernoulli and ordinal nodes were modeled using logit links; the survival node used a Weibull proportional hazards specification with age as the analysis time scale. Estimates are reported as β coefficients for Gaussian equations, odds ratios (ORs) for Bernoulli and ordinal logit equations, and hazard ratios (HRs) for the Weibull survival equation; raw coefficients and standard errors are retained for transparency

### Sensitivity analyses

Sensitivity analyses across competing-risk regression, multistate models, and network-based approaches yielded a coherent pattern. In competing-risk models accounting for death, frailty was associated with higher risk of incident cognitive impairment in minimally adjusted models, but these associations attenuated and were no longer statistically significant after full adjustment; HEI-2015 total score was not associated with composite cognitive outcomes. Multistate models showed similar attenuation for the transition from normal cognition to MCI/CIND, while frailty remained strongly associated with mortality transitions, particularly from MCI/CIND to death. HEI-2015 showed limited associations, with a modest inverse relationship observed for transitions from normal cognition to death (Supplementary Materials 3:Supplementary datasheet 7 and Figure S3, Appendices and *Github*).

Network-based analyses provided complementary insights. In reduced ABN models, the cognitive outcome was directly linked to age, race/ethnicity, and frailty, with HEI-2015 operating upstream through socioeconomic and behavioral pathways. These reduced-network findings are consistent with a possible indirect pathway linking diet quality, frailty, and cognition, but the pathway was sensitive to network specification and should not be interpreted as evidence of causal mediation. Generalized structural equation models (GSEM) derived from ABN confirmed that frailty retained modest direct association with incident cognitive impairment, while HEI-2015 was not directly associated with this outcome. Together, these findings suggest that frailty may reflect a broader vulnerability phenotype associated with socioeconomic and lifestyle factors, while the relationship between diet quality and cognition may partly overlap with or operate through pathways related to frailty rather than representing a strong independent association (Supplementary Materials 3:Supplementary datasheet 8 and Supplementary Materials 2:Figure S4, Appendices and *Github*).

## Discussion

In this sample of older U.S. adults drawn from the nationally representative Health and Retirement Study, higher overall diet quality was consistently associated with lower frailty, whereas overall HEI-2015 was not independently associated with incident cognitive impairment. Exploratory analyses of individual food items identified heterogeneous associations with the composite CIND/probable dementia outcome after comprehensive adjustment. Higher consumption of selected dairy products, processed meats, fortified orange juice, certain fruits, and sweetened or low-calorie beverages was associated with higher cognitive impairment risk, whereas other cheese and red wine showed inverse associations. The strongest nominal inverse association was observed for red wine, although this finding should not be interpreted as evidence of a protective effect of alcohol consumption. Importantly, none of the food-item associations with composite cognitive impairment or MCI/CIND alone survived FDR correction, indicating that these findings may reflect chance associations arising from multiple testing. Five associations with probable dementia alone survived FDR correction, although these remained exploratory because of limited event counts and potential residual confounding. Four-way decomposition analyses suggested that the controlled direct effect of frailty predominated, with little evidence of mediation or interaction involving overall diet quality, although selected food-specific interactions were observed. Frailty was not a direct predictor of cognitive impairment in the primary ABN-informed GSEM, despite a modest direct association in the reduced model. Collectively, these findings support a consistent association between diet quality and physical frailty but more limited and heterogeneous evidence linking specific dietary exposures or frailty independently to cognitive impairment.

Prior literature consistently supports an inverse association between healthy dietary patterns and frailty, and our findings align with this well-established evidence base, such as in InCHIANTI study [13] and Osteoporotic Fractures in Men Study [14] and more recent meta-analyses and pooled analyses [39, 40]. Dietary food groups have also been reported to be associated with frailty. In the Nurses’ Health Study of older women, higher plant protein intake was associated with lower frailty risk, whereas higher animal protein intake was associated with higher frailty risk [41]. A recent systematic review and meta-analysis including 15 studies and 42,130 study participants found that eating a pro-inflammatory diet, as measured using the Dietary Inflammation Index, increased the risk of both pre-frailty and frailty[42].

Our findings help integrate these bodies of literature by showing that diet quality, frailty, and cognition are interrelated within a broader network of socioeconomic, behavioral, and health-related factors. Diet was strongly associated with frailty, whereas the frailty–cognition association attenuated after comprehensive adjustment, suggesting that frailty may partly reflect shared vulnerability rather than acting as an independent proximal determinant of cognitive impairment. In fact, a large body of evidence shows that frailty is strongly associated with cognitive decline and dementia [39, 43, 44], with meta-analyses further demonstrating that cognitive frailty, a combined phenotype of physical frailty and cognitive impairment, confers particularly high risk of adverse outcomes, including mortality [45, 46].

Our complementary analyses consistently linked overall diet quality, measured by the HEI-2015 total score, with lower frailty but found limited evidence of an independent association with incident cognitive impairment [47, 48]. Exploratory analyses of the 13 HEI-2015 component scores and individual HCNS food and beverage items revealed heterogeneous associations with cognitive outcomes. Although several individual dietary items showed nominal associations, none remained significant after FDR correction for the composite CIND/probable dementia or MCI/CIND-only outcomes. Five associations remained for probable dementia alone, but these findings require cautious interpretation and independent replication given the limited number of events and imprecision of some estimates. Accordingly, our findings support further investigation of HEI components and individual food and beverage items without establishing that these measures are more informative for cognitive aging than overall diet quality.

Our findings suggest that frailty and cognitive impairment share upstream aging mechanisms rather than occurring sequentially. Frailty predicts cognitive decline, dementia, and mortality, with death often competing with progression to cognitive impairment. Robertson et al. proposed that chronic inflammation, vascular dysfunction, sarcopenia, and multisystem physiological dysregulation may jointly underlie both frailty and cognitive decline [43], while Rogers et al. (2017) demonstrated that frailty predicts incident dementia independent of chronological age, although attenuation after extensive adjustment suggests partial confounding by shared determinants [10]. More recent epidemiologic studies further support strong cross-sectional and longitudinal links between frailty and poorer cognitive performance across multiple domains, including memory, executive function, processing speed, and global cognition [49, 50]. Collectively, these findings suggest that frailty reflects broader physiological, psychological, and social vulnerability that may influence cognitive decline through multiple pathways rather than solely as a mediator.

Healthier diets rich in fruits, vegetables, legumes, fish, nuts, and whole grains are associated with lower cognitive decline and dementia risk, whereas pro-inflammatory and highly processed diets show adverse associations [4]. Recent evidence from systematic reviews and meta-analyses further supports protective associations for Mediterranean-style, MIND, and other plant-forward dietary patterns with cognitive resilience, slower cognitive decline, and lower dementia risk [51–53]. Randomized dietary intervention studies additionally suggest that Mediterranean-style dietary interventions may improve global cognition, MMSE/CASI performance, and ADAS-Cog scores among older adults [54]. Although some studies have linked higher overall diet quality, including HEI-2015, to lower dementia risk or better cognitive performance [55, 56], findings vary across cohorts and adjustment strategies. Mediterranean diet alignment has been associated with lower dementia risk and better domain-specific cognition [48, 57], and meta-analyses generally support healthier dietary patterns [58]. However, other studies report attenuated or null associations, reflecting differences in dietary assessment, follow-up, and outcome definitions [47, 59]. Pro-inflammatory diets have also been associated with cognitive impairment [60] and dementia, particularly among older adults with cardiometabolic diseases [61] and older women [62]. Thus, specific dietary food groups and inflammatory potential may be more informative than overall diet indices.

Several studies additionally suggest that individual food groups may contribute more strongly to cognitive aging than composite dietary indices alone. For example, higher processed red meat intake has been associated with increased dementia risk, whereas unprocessed red meat often shows weaker or null associations [63]. Chronic blueberry supplementation, berry intake, and flavonoid-rich dietary patterns have been associated with slower cognitive decline and lower dementia risk, particularly in domains related to episodic memory and global cognition [64–66]. Greater fruit and vegetable intake has likewise been linked to better cognitive trajectories and slower decline across both U.S. and Chinese cohorts [67]. In contrast, higher ultra-processed food consumption has been associated with accelerated cognitive decline [68], while artificially sweetened soft drinks and non-wine alcohol intake have been linked to less favorable cognitive outcomes or higher dementia risk [69, 70]. Moreover, evidence from dairy studies suggests that subtype and fat content may differentially relate to cognition, with high-fat cheese and cream showing inverse associations with dementia risk while total dairy intake obscures these component-specific effects [71, 72]. The inverse association between red wine intake and incident cognitive impairment (HR = 0.77, 95% CI: 0.66–0.89) warrants cautious interpretation. Previous observational studies have suggested that wine consumption may be associated with lower dementia risk, potentially reflecting polyphenol-related antioxidant and vascular mechanisms [70, 73]. However, residual confounding, differences between drinkers and abstainers, and reverse causation may explain these associations. Recent evidence also challenges the presumed cognitive benefits of moderate alcohol consumption [74]. Importantly, our association did not survive FDR correction and should not be interpreted as supporting alcohol consumption for dementia prevention.

Emerging evidence suggests that diet may influence cognitive aging through pathways involving physical frailty and broader nutritional vulnerability. Mediterranean-style dietary patterns have been associated with lower incident frailty[39, 75], whereas declining dietary diversity and malnutrition have been linked to cognitive frailty, a syndrome characterized by coexisting physical frailty and cognitive impairment [76, 77]. Our findings are consistent with this literature in demonstrating a persistent inverse association between overall diet quality and physical frailty. However, we found limited evidence that overall diet quality was independently associated with incident cognitive impairment or that dietary factors substantially modified the frailty–cognition association. Furthermore, the attenuation of frailty–cognition associations following comprehensive adjustment suggests that socioeconomic, behavioral, and clinical characteristics may contribute to these relationships. Thus, our results extend previous research by distinguishing the relatively consistent diet–frailty association from the more complex and potentially confounded relationships involving cognitive impairment. These findings support viewing frailty as a broader marker of nutritional and physiological vulnerability rather than establishing it as a causal mediator of dietary influences on cognitive aging. Further details are provided in Supplementary Materials 3:Supplementary Datasheet 9 and Supplementary Materials 1: Appendix H.

Strengths include the use of data from the large, nationally representative, longitudinal HRS cohort, which provides detailed sociodemographic, behavioral, clinical, dietary, functional, and cognitive information on older U.S. adults. The temporal sequence of the principal measures—diet in 2013, frailty and baseline cognition in 2016, and cognitive follow-up through 2022—permitted prospective evaluation of incident cognitive impairment. We used established measures, including the HEI-2015, a modified Fried frailty phenotype, and the validated Langa–Weir algorithm for cognitive classification. Analyses incorporated survey weights, clustering, and stratification to account for the complex sampling design. Sequential covariate adjustment and complementary approaches—including Cox regression, competing-risk and multistate models, four-way decomposition, additive Bayesian networks, and generalized structural equation modeling—allowed us to evaluate the consistency of findings across different assumptions and characterize direct, indirect, and interrelated pathways among diet, frailty, and cognition. Examination of individual dietary food groups also provided greater detail than analyses limited to overall diet quality.

Several limitations should be considered. Diet was assessed once using the self-reported 2013 HCNS food-frequency questionnaire and assumed to represent exposure before frailty assessment in 2016 and cognitive follow-up during 2018–2022. Unmeasured dietary changes related to aging, illness, functional limitations, or preclinical cognitive decline may have caused exposure misclassification, attenuation, or reverse-causation bias. The modest sample limited power, particularly for probable dementia, less frequently consumed foods, and sex-specific analyses. Gender identity and related social influences were not assessed. Clinical relevance also warrants caution: each 1-SD higher HEI-2015 score corresponded to only a 0.13-point lower frailty score on the 0–5 scale, while frailty–cognition associations attenuated after full adjustment and overall HEI-2015 was unrelated to cognition. Hazard ratios represent relative, not absolute, risk and should not be interpreted as evidence of substantial clinical benefit. Cognition was classified using the validated Langa–Weir algorithm, but MMSE, MoCA, and domain-specific outcomes were unavailable, potentially obscuring associations with specific cognitive functions. Frailty was based on available survey and performance measures rather than a complete clinical Fried assessment. Although HRS is nationally representative, the HCNS-linked complete-case sample may not be; HCNS weights cannot fully address selection from missing data or attrition, limiting generalizability. Residual confounding and the observational design preclude causal inference. Future studies should use repeated dietary and domain-specific cognitive assessments, larger samples, and trajectory, time-varying exposure, joint longitudinal-survival, or measurement-error models. Randomized nutritional interventions are ultimately needed to determine whether sustained dietary modification produces clinically meaningful cognitive benefits.

## Conclusions

In summary, higher overall diet quality was consistently associated with lower physical frailty, whereas overall HEI-2015 was not independently associated with incident cognitive impairment. Exploratory analyses identified heterogeneous associations between individual food items and cognitive outcomes, including nominally higher risks associated with selected dairy products, processed meats, and beverages, and lower risks associated with other cheese and red wine. However, none of the associations with composite CIND/probable dementia or MCI/CIND alone survived FDR correction, limiting their interpretation. Although selected associations with probable dementia alone remained significant after FDR correction, these findings require cautious interpretation and independent replication. Four-way decomposition analyses provided limited evidence of dietary mediation or broad effect modification of the frailty–cognition relationship. Overall, the findings support the importance of diet quality in relation to physical frailty while highlighting the need for further research to clarify food-specific associations with cognitive aging.

## Supplementary information

Supplementary material 1: Appendices A-H

Supplementary material 2: Figs. S1-S4

Supplementary material 3: Supplementary datasheets 1-9

Supplementary material 4: Survey-weighted baseline characteristics of participants by HEI-2015 categories and frailty status, Health and Retirement Study (2016)

Supplementary material 5: List of supplementary materials

## Data Availability

HRS data are publicly available at: https://hrs.isr.umich.edu/about. Code and Output can be obtained from the corresponding author at baydounm@mail.nih.gov and will be made available on github at: https://github.com/baydounm/HRS_DIET_FRAIL_COGN_FINAL.
