# Supplementary material for: Prospective associations between diet quality, frailty and incident cognitive impairment in the health and retirement study
Source: BMC Glob Public Health. 2026 Nov 11;4:109. doi: 10.1186/s44263-026-00347-x (PMC13753220; doi:10.1186/s44263-026-00347-x)
Supplement: Supplementary file 1 [file 44263_2026_347_MOESM1_ESM.pdf]

Supplementary Materials 1

Prospective Associations between Diet Quality, Frailty and Incident Cognitive Impairment in the Health and Retirement Study

General Description

Appendix A describes data sources, eligibility, variable construction, and survey design; Appendix B describes the statistical analyses. Subsequent appendices present the four-way decomposition, network and structural analyses, sensitivity analyses, and reproducibility information. Supplementary Datasheets 1–8 provide the corresponding codebooks and numerical results; Supplementary Datasheet 9 provides the supporting literature review.

Appendix A. Data sources and variable construction

The analysis linked HRS covariates measured primarily in 2012, dietary information from the 2013 Health Care and Nutrition Study, frailty and baseline cognition assessed in 2016, and cognitive follow-up in 2018, 2020, and 2022. Participants were aged 50 years or older, interviewed in 2016, cognitively normal at baseline, and had complete diet, frailty, covariate, and survey-design information. The final analytic sample included 1,636 participants.

Analyses used the HCNS sampling weight, HRS strata, and primary sampling units. Continuous diet variables were standardized within the analytic sample so that estimates represent a 1-SD increment. The HEI-2015 total score ranged from 0 to 100. Fixed categories of <51, 51–80, and ≥81 were used for descriptive analyses; sample-specific quartiles were used for Kaplan–Meier plots and secondary categorical survival analyses.

The Health and Retirement Study is a nationally representative longitudinal cohort of U.S. adults aged 50 years or older with biennial follow-up. This analysis linked dietary data from the 2013 Health Care and Nutrition Study, covariates measured primarily in 2012, diet in 2013, frailty and baseline cognition in 2016, and longitudinal cognitive assessments through 2022. Eligible participants were required to have valid dietary data, frailty data, covariates, and normal cognition at baseline. The final analytic sample was restricted to community-dwelling participants with complete data and valid survey design variables, and analyses applied HCNS sampling weights with strata and primary sampling units. A generic survey-weighted estimand for a mean outcome Y can be written as $\hat{\mu}_w = \frac{\sum_{i=1}^{n} w_i Y_i}{\sum_{i=1}^{n} w_i}$, where w_i denotes the survey weight for participant i. In Stata, the survey design can be specified as:

```
use "HRS_HEI_FRAIL_ANALYTIC_FINALfin.dta", clear
keep if sample_final==1
svyset secu [pweight=HCNSWGTR_NT], strata(stratum) singleunit(centered)
```

Diet quality was assessed using the Healthy Eating Index–2015 derived from HCNS food frequency questionnaire data. Let C_{ij} denote participant i's score for dietary component j, where $j = 1, \dots, 13$. The total HEI score was computed as $HEI_i = \sum_{j=1}^{13} C_{ij}$, with $0 \le HEI_i \le 100$. Component scores reflected adequacy domains, for which higher intake is favorable, and moderation domains, for which lower intake is favorable. For modeling, the total HEI was standardized as $zHEI_i = \frac{HEI_i - \bar{HEI}}{SD(HEI)}$. For descriptive analyses, HEI was categorized using fixed cutoffs (<51, 51–80, and ≥81); sample-specific quartiles were generated separately for Kaplan–Meier plots and secondary categorical survival analyses. Example Stata code is:

```
* Standardized score for primary regression analyses
egen zhei2015_total_score = std(hei2015_total_score)

* Fixed categories for descriptive analyses
gen hei_desc = .
replace hei_desc = 1 if hei2015_total_score < 51
```

```
replace hei_desc = 2 if hei2015_total_score >= 51 & hei2015_total_score < 81
replace hei_desc = 3 if hei2015_total_score >= 81 & hei2015_total_score < .
label define hei_desc 1 "Poor (<51)" 2 "Needs improvement (51-80)" 3 "Good (>=81)"
label values hei_desc hei_desc

* Sample-specific quartiles for Kaplan-Meier and secondary survival analyses
xtile hei_quartile = hei2015_total_score, nq(4)
```

Exploratory analyses also evaluated individual HEI components and grouped food variables after standardization:

```
foreach v of varlist heix* C3A_FF_13-C9AP_FF_13 {
   egen z`v' = std(`v')
}
```

Frailty was defined using a modified Fried phenotype based on five binary criteria: weight loss, weakness, exhaustion, slowness, and low physical activity. Let $F_{ik} \in [0,1]$ denote the presence of frailty criterion k for individual i, where $k = 1, \ldots, 5$. The frailty score was $Frailty_i = \sum_{k=1}^{5} F_{ik}$, yielding a range from 0 to 5. Frailty status was then categorized as robust if $Frailty_i = 0$, pre-frail if $Frailty_i \in [1,2]$, and frail if $Frailty_i \geq 3$. All five component values were required to calculate the frailty score. Example Stata code is:

```
egen n_frailty_nonmiss = rownonmiss(weightloss weakness exhaustion slowness lowpa)
egen fried_sum = rowtotal(weightloss weakness exhaustion slowness lowpa)
replace fried_sum = . if n_frailty_nonmiss < 5

gen fried_status = .
replace fried_status = 0 if fried_sum == 0
replace fried_status = 1 if inrange(fried_sum, 1, 2)
replace fried_status = 2 if fried_sum >= 3 & fried_sum < .
label define fried 0 "Robust" 1 "Pre-frail" 2 "Frail"
label values fried_status fried
```

For some analyses, an alternative three-level variable was generated:

```
gen frail_cat = .
replace frail_cat = 1 if fried_sum==0
replace frail_cat = 2 if inrange(fried_sum,1,2)
replace frail_cat = 3 if fried_sum>=3 & fried_sum<.
label define frail_cat 1 "Robust (0)" 2 "Pre-frail (1-2)" 3 "Frail (>=3)"
label values frail_cat frail_cat
```

Incident cognitive impairment was defined using the Langa–Weir algorithm, which classifies participants as normal cognition, cognitively impaired non-dementia, or probable dementia based on cognitive testing for self-respondents and proxy-based information for proxy respondents. Participants cognitively normal at baseline were followed to incident CIND or probable dementia, with outcomes defined as composite CIND/probable dementia, CIND-only, and probable-dementia-only. In time-to-event notation, for each participant i, observed follow-up time was $T_i = \min(T_i^*, C_i)$, where T_i^* is the true event time and C_i is the censoring time. Event indicators were defined separately for the composite, CIND-only, and probable-dementia-only outcomes. Example Stata survival setup code is:

```
stset ageevent, origin(time ageenter) enter(time ageenter) ///
   failure(fail_cog==1) id(HHIDPN)

stset doevent_mci, origin(time doenter) enter(time doenter) ///
   failure(fail_mci==1) id(HHIDPN)

stset doevent_dem, origin(time doenter) enter(time doenter) ///
   failure(fail_dem==1) id(HHIDPN)
```

Covariates were selected a priori and included age, sex, race/ethnicity, education, total household income, marital status, smoking, alcohol use, physical activity, body mass index, hypertension, diabetes, stroke, heart disease, and self-rated health. A principal component–based socioeconomic status index was also used in some modeling frameworks. Let X_i denote the vector of covariates for participant i. Sequential models were fitted to evaluate attenuation of associations after adjustment for progressively richer sets of confounders. Example Stata global macros aligned with the analytic workflow are:

```
global M1 "c.r13agey_e i.ragender"
global M2 "c.r13agey_e i.ragender i.RACE_ETHN i.marital_2012 i.education i.income_cat_2012"
global M3 "c.r13agey_e i.ragender i.RACE_ETHN i.marital_2012 i.education i.income_cat_2012 ///
        i.smoking_2012 i.alcohol_2012 i.physic_act_2012 i.bmibr_2012 ///
        i.hbp_ever_2012 i.diab_ever_2012 i.stroke_ever_2012 i.heart_ever_2012 i.srh_2012"
```

A principal component index can be constructed as:

```
egen z_education = std(education)
egen z_income_cat_2012 = std(income_cat_2012)
pca z_education z_income_cat_2012
predict ses_pc1, score
egen ses_index = std(ses_pc1)
```

Appendix B. Statistical analysis

Baseline characteristics were estimated using survey-weighted means and proportions. For continuous variables, group comparisons were based on design-adjusted Wald tests from survey-weighted linear regression. For categorical variables, design-adjusted Rao–Scott or Pearson tests were used. If Y_i is continuous and G_i is a categorical grouping variable, the descriptive comparison model can be written as $Y_i = \alpha_0 + \sum_{m=1}^{M-1} \alpha_m I(G_i = m) + \epsilon_i$. Example Stata code is:

```
svy: mean r13agey_e hei2015_total_score fried_sum
svy: mean r13agey_e, over(hei_desc)
svy: mean r13agey_e, over(fried_status)

svy: tab hei_desc ragender, row percent pearson
svy: tab fried_status ragender, row percent pearson

svy: regress r13agey_e i.hei_desc
testparm i.hei_desc
```

Associations between diet quality and frailty were evaluated using survey-weighted linear regression. The core model was $Frailty_i = \beta_0 + \beta_1 zHEI_i + X_i^{\top}\beta + \varepsilon_i$, where β_1 estimates the mean difference in frailty score per one standard deviation higher HEI-2015. The same framework was also applied to individual HEI components and food groups. Example Stata code is:

```
svy: regress fried_sum c.zhei2015_total_score $M1
lincom c.zhei2015_total_score

svy: regress fried_sum c.zhei2015_total_score $M2
lincom c.zhei2015_total_score

svy: regress fried_sum c.zhei2015_total_score $M3
lincom c.zhei2015_total_score

foreach x of varlist zheix* zC3A_FF_13-zC9AP_FF_13 {
   svy: regress fried_sum c.`x' $M3
   lincom c.`x'
}
```

Prospective associations with incident cognitive impairment were evaluated using survey-weighted Cox proportional hazards models. For participant i, the hazard at time t was modeled as $h_i(t) =$

$h_0(t)\exp(\beta_1 Frailty_i + \beta_2 HEI_i + X_i^\top \beta)$, where $h_0(t)$is the unspecified baseline hazard. Frailty was modeled both categorically and continuously, depending on the analysis. Example Stata code for categorical frailty is:

```
stset ageevent, origin(time ageenter) enter(time ageenter) ///
   failure(fail_cog==1) id(HHIDPN)

svy: stcox i.frail_cat $M1
svy: stcox i.frail_cat $M2
svy: stcox i.frail_cat $M3
```

Example code for diet predicting cognition, with frailty included as a covariate, is:

```
global M3diet "c.r13agey_e i.ragender i.RACE_ETHN i.marital_2012 i.education i.income_cat_2012 ///
        i.smoking_2012 i.alcohol_2012 i.physic_act_2012 i.bmibr_2012 ///
        i.hbp_ever_2012 i.diab_ever_2012 i.stroke_ever_2012 i.heart_ever_2012 ///
        i.srh_2012 i.frail_cat"

svy: stcox c.zhei2015_total_score $M3diet
```

Appendix C. Four-way decomposition

Four-way decomposition was conducted in Stata using the med4way procedure as an exploratory analysis of the joint roles of frailty and diet in incident cognitive impairment. The continuous Fried frailty score was specified as the exposure, and each standardized dietary measure was entered separately as the mediator and effect modifier. Separate analyses were conducted for incident CIND/probable dementia, CIND-only, and probable-dementia-only outcomes.

For each outcome, three covariate specifications were estimated. The fully adjusted model ($COV4) was the primary model; the reduced model ($COVS2) and age- and sex-adjusted model ($COVS2_AGESEX) were supplementary. The Cox outcome model included frailty, diet, their interaction, and the covariates defined for the applicable specification; the mediator model was linear. Frailty contrasts were set at 0 and 1, and the dietary reference value was the sample median. Frailty was selected as the exposure because the frailty–cognition total association was stronger and more consistently nominally significant than the diet–cognition association. Decompositions were nevertheless estimated for all three cognitive outcomes under all three adjustment specifications. Because diet was measured before frailty, mediation components are interpreted descriptively and not as evidence that frailty caused subsequent dietary change; interaction components are interpreted as exploratory assessments of whether diet modifies the frailty–cognition association. Complete estimates and convergence indicators are reported in Supplementary Datasheet 4.

Counterfactual-based four-way decomposition was used to partition the total effect of an exposure Aon outcome Ythrough a mediator Minto controlled direct effect, reference interaction, mediated interaction, and pure indirect effect. This can be written as $TE = CDE + INT_{ref} + INT_{med} + PIE$. The mediator model was specified as $M_i = \alpha_0 + \alpha_1 A_i + X_i^\top \alpha + \epsilon_i$, and the outcome model included both main effects and the exposure-by-mediator interaction, for example $h_i(t) = h_0(t)\exp(\gamma_1 A_i + \gamma_2 M_i + \gamma_3 A_i M_i + X_i^\top \gamma)$under a Cox model. Example Stata code, aligned with the workflow in which frailty score was the exposure and HEI was the mediator/effect modifier, is:

```
* Example med4way command used within the loop
* Exposure: frailty score
* Mediator/effect modifier: standardized HEI-2015 total score
* Outcome: Cox model after stset
* Covariates: primary fully adjusted specification ($COV4)

stset doevent, origin(time doenter) enter(time doenter) ///
```

```
    failure(fail_cog==1) id(HHIDPN)

quietly summarize zhei2015_total_score if zhei2015_total_score < ., detail
local m0 = r(p50)

med4way fried_sum zhei2015_total_score $COV4, ///
    a0(0) a1(1) m(`m0') ///
    yreg(cox) mreg(linear) ///
fulloutput

* Parallel models: composite CIND/probable dementia, CIND-only, and probable-dementia-only
* Adjustment sets: $COV4 (primary), $COVS2 and $COVS2_AGESEX (supplementary)
```

Core equations for the four-way decomposition are given below.

A= frailty exposure

M= diet quality or a dietary component

Y= incident cognitive impairment

$\mathbf{C}$= covariates

1. Mediator model

Because diet quality/components were modeled as continuous mediators, the mediator model is:

$$M_i = \alpha_0 + \alpha_1 A_i + \mathbf{C}_i^{\top} \boldsymbol{\alpha} + \varepsilon_i$$

So the expected mediator under exposure level $a$is:

$$E(M_a \mid \mathbf{C}) = \alpha_0 + \alpha_1 a + \mathbf{C}^{\top} \boldsymbol{\alpha}$$

and under the reference exposure a^*:

$$E(M_{a^*} \mid \mathbf{C}) = \alpha_0 + \alpha_1 a^* + \mathbf{C}^{\top} \boldsymbol{\alpha}$$

Hence the exposure-induced change in the mediator is:

$$E(M_a \mid \mathbf{C}) - E(M_{a^*} \mid \mathbf{C}) = \alpha_1 (a - a^*)$$

2. Outcome model

For the Cox model with an exposure–mediator interaction, the hazard is written as:

$$h(t \mid A, M, \mathbf{C}) = h_0(t) \exp(\beta_1 A + \beta_2 M + \beta_3 AM + \mathbf{C}^{\top} \boldsymbol{\beta}_c)$$

Equivalently, on the log-hazard scale:

$$\log h(t \mid A, M, \mathbf{C}) = \log h_0(t) + \beta_1 A + \beta_2 M + \beta_3 AM + \mathbf{C}^{\top} \boldsymbol{\beta}_c$$

Here:

β_1= main effect of frailty

β_2= main effect of diet

β_3= frailty × diet interaction

3. Counterfactual total effect

Let $a$be the observed exposure level and $a^*$the reference exposure level.

The total effect is the contrast:

$$TE = Y_{a,M_a} - Y_{a^*,M_{a^*}}$$

In the decomposition framework, this total effect is partitioned into four components:

$$TE = CDE + INT_{ref} + INT_{med} + PIE$$

where:

CDE= controlled direct effect

INT_{ref}= reference interaction

INT_{med}= mediated interaction

PIE= pure indirect effect

4. Controlled direct effect

The controlled direct effect is the effect of changing frailty from $a^*$to $a$while holding the mediator fixed at a chosen value m:

$$CDE(m) = Y_{a,m} - Y_{a^*,m}$$

Under the Cox model, on the linear predictor or log-hazard scale:

$$CDE(m) = (\beta_1 + \beta_3 m)(a - a^*)$$

and on the hazard ratio scale:

$$HR_{CDE}(m) = \exp[(\beta_1 + \beta_3 m)(a - a^*)]$$

5. Reference interaction

The reference interaction captures effect modification by the mediator, independent of mediation. It is the part due to the interaction between frailty and diet, evaluated at the mediator level that would arise under the reference exposure:

$$INT_{ref} = Y_{a,M_{a^*}} - Y_{a,m} - Y_{a^*,M_{a^*}} + Y_{a^*,m}$$

Using the linear mediator model, on the log-hazard scale:

$$INT_{ref} = \beta_3(a - a^*)[E(M_{a^*} \mid \mathbf{C}) - m]$$

Since

$$E(M_{a^*} \mid \mathbf{C}) = \alpha_0 + \alpha_1 a^* + \mathbf{C}^\top \boldsymbol{\alpha},$$

this becomes:

$$INT_{ref} = \beta_3(a - a^*)(\alpha_0 + \alpha_1 a^* + \mathbf{C}^\top \boldsymbol{\alpha} - m)$$

and on the hazard ratio scale:

$$HR_{INT_{ref}} = \exp\{\beta_3(a - a^*)[E(M_{a^*} \mid \mathbf{C}) - m]\}$$

6. Mediated interaction

The mediated interaction is the portion due jointly to mediation and exposure–mediator interaction:

$$INT_{med} = Y_{a,M_a} - Y_{a,M_{a^*}} - Y_{a^*,M_a} + Y_{a^*,M_{a^*}}$$

Under the linear mediator model, on the log-hazard scale:

$$INT_{med} = \beta_3(a - a^*)[E(M_a \mid \mathbf{C}) - E(M_{a^*} \mid \mathbf{C})]$$

Since

$$E(M_a \mid \mathbf{C}) - E(M_{a^*} \mid \mathbf{C}) = \alpha_1(a - a^*),$$

this simplifies to:

$$INT_{med} = \beta_3\alpha_1(a - a^*)^2$$

and on the hazard ratio scale:

$$HR_{INT_{med}} = \exp[\beta_3\alpha_1(a - a^*)^2]$$

7. Pure indirect effect

The pure indirect effect is the portion due to mediation alone, in the absence of interaction:

$$PIE = Y_{a^*,M_a} - Y_{a^*,M_{a^*}}$$

Under the Cox model, on the log-hazard scale:

$$PIE = \beta_2[E(M_a \mid \mathbf{C}) - E(M_{a^*} \mid \mathbf{C})]$$

Substituting the linear mediator model:

$$PIE = \beta_2\alpha_1(a - a^*)$$

and on the hazard ratio scale:

$$HR_{PIE} = \exp[\beta_2\alpha_1(a - a^*)]$$

8. Total effect from the four components

Adding the four pieces:

$$TE = CDE + INT_{ref} + INT_{med} + PIE$$

Substituting the expressions above on the log-hazard scale:

$$\begin{aligned} TE = (\beta_1{+}\beta_3 m)(a - a^*) + \beta_3(a - a^*)[E(M_{a^*} \mid \mathbf{C}) - m] + \beta_3(a - a^*)[E(M_a \mid \mathbf{C}) - E(M_{a^*} \mid \mathbf{C})] \\ + \beta_2[E(M_a \mid \mathbf{C}) - E(M_{a^*} \mid \mathbf{C})] \end{aligned}$$

This reduces to:

$$TE = \beta_1(a - a^*) + \beta_2\alpha_1(a - a^*) + \beta_3(a - a^*)E(M_a \mid \mathbf{C})$$

and on the hazard ratio scale:

$$HR_{TE} = \exp(TE)$$

9. Derived quantities often reported by med4way

These are often summarized from the four basic pieces.

Overall mediated effect

$$Med = PIE + INT_{med}$$

Overall interaction effect

$$Int = INT_{ref} + INT_{med}$$

Proportion mediated

$$Prop_{med} = \frac{PIE + INT_{med}}{TE}$$

Proportion due to interaction

$$Prop_{int} = \frac{INT_{ref} + INT_{med}}{TE}$$

Proportion eliminated

This is the proportion of the total effect that would be eliminated if the mediator were fixed at m:

$$Prop_{elim} = \frac{INT_{ref} + INT_{med} + PIE}{TE}$$

10. Natural direct and indirect effects

These can also be derived from the four-way decomposition.

Pure natural direct effect

$$PNDE = CDE + INT_{ref}$$

Total natural indirect effect

$$TNIE = PIE + INT_{med}$$

So:

$$TE = PNDE + TNIE$$

11. Current setup for analysis

Frailty was the exposure, diet quality or an individual dietary measure was the mediator/effect modifier, and incident cognitive impairment was the outcome. Thus, the models become:

Mediator model

$$Diet_i = \alpha_0 + \alpha_1 Frailty_i + \mathbf{C}_i^{\top}\boldsymbol{\alpha} + \varepsilon_i$$

Outcome model

$$h(t \mid Frailty, Diet, \mathbf{C}) = h_0(t)\exp(\beta_1 Frailty + \beta_2 Diet + \beta_3(Frailty \times Diet) + \mathbf{C}^{\top}\boldsymbol{\beta}_c)$$

Then:

$$PIE = \beta_2\alpha_1(a - a^*)$$
$$INT_{med} = \beta_3\alpha_1(a - a^*)^2$$
$$INT_{ref} = \beta_3(a - a^*)[E(Diet_{a^*} \mid \mathbf{C}) - m]$$
$$CDE = (\beta_1 + \beta_3 m)(a - a^*)$$

Sources: [1]

Appendix D. Additive Bayesian network analysis

Additive Bayesian networks were estimated in R using complete cases. The models included demographic characteristics, SES, marital status, lifestyle and health variables, diet, frailty, time indicators, and the cognitive outcome. Variables were represented as Gaussian or binomial nodes according to their coding. Candidate networks were compared using a score-based hill-climbing search with Bayesian information criterion scoring and parent limits.

Directional restrictions were imposed so that the cognitive outcome had no outgoing arcs and diet preceded frailty. Demographic and socioeconomic characteristics were treated as upstream variables. The network was used to identify conditional dependencies; its arcs were not interpreted as definitive causal effects. Retained arcs and estimates are provided in Supplementary Datasheet 5.

Additive Bayesian networks (ABN) were used to identify conditional dependency structures among diet quality, frailty, cognition, and upstream demographic, socioeconomic, behavioral, and clinical factors. Let $V = (V_1, \dots, V_p)$ denote the set of variables in the network. Under a directed acyclic graph G, the joint distribution factorizes as

$$P(V) = \prod_{j=1}^{p} P(V_j \mid \mathrm{Pa}(V_j)),$$

where $\mathrm{Pa}(V_j)$denotes the parent set of node V_j.

Model selection was based on maximizing a penalized likelihood score, specifically the Bayesian Information Criterion,

$$\mathrm{BIC}(G) = \log L(G) - \frac{k_G}{2}\log(n),$$

where $L(G)$is the likelihood under graph G, $k_G$is the number of free parameters, and $n$is the sample size.

Variables were specified according to their scale. Continuous variables, such as age, socioeconomic status, HEI-2015 total score, and frailty score, were modeled using Gaussian distributions with identity links. Binary variables were modeled using Bernoulli distributions with logit links, and ordinal variables using ordinal logistic distributions. The cognitive outcome was treated as a terminal node.

To ensure biologically and temporally plausible structures, arcs were constrained according to the following ordering:

Demographics → Socioeconomic factors → Behavioral factors and diet → Clinical conditions → Frailty → Cognitive outcome.

Demographic variables were treated as exogenous. Socioeconomic variables were allowed to depend on demographics but not on downstream nodes. Behavioral variables and diet quality were allowed to depend on demographic and socioeconomic variables. Clinical conditions were permitted to depend on upstream determinants. Frailty was modeled as a downstream node influenced by behavioral and clinical factors. The cognitive outcome was constrained to be terminal, with no outgoing edges.

The parent limit was set to allow up to four parents per node for all non-outcome variables. The cognitive outcome node was handled as a special case: in addition to up to four substantive parents, it was allowed to include the two time indicators $t2$and $t3$, yielding an effective maximum of four plus two parents for the outcome. This specification preserved the longitudinal structure of the discrete-time cognitive outcome model while maintaining comparable complexity constraints across the rest of the network. Additional banned arcs were imposed to prevent reverse causation and to enforce temporal consistency, such as disallowing cognition as a parent of upstream variables.

ABN models were estimated in R using the *abn* package. A score-based approach was implemented by first constructing a score cache of candidate parent sets under the specified constraints, followed by hill-climbing search:

```
library(abn)

score.cache <- buildScoreCache(
  data.df     = dat,
  data.dists  = dists,
  dag.banned  = banned.mat,
  max.parents = 4
)

dag <- searchHillClimber(score.cache)
plotAbn(dag$dag)
```

Because the cognitive outcome was permitted to exceed the general parent limit through inclusion of $t2$and $t3$, the final search specification treated the outcome as an exception to the four-parent rule. Thus, the general network complexity constraint was four parents per child, whereas the outcome could retain two additional time-dummy parents when supported by the scoring procedure.

The final ABN structure was then used to inform generalized structural equation models (GSEM). In the GSEM stage, the time dummies were omitted by design, and continuous variables were retained in their original form rather than discretized. The cognitive outcome was modeled using a Weibull survival specification with age as the analysis time scale. Accordingly, the ABN and GSEM stages are complementary: ABN was used for data-driven structure learning under discrete-time constraints, whereas GSEM quantified the corresponding pathways in a continuous-variable survival framework.

Edges in the ABN represent conditional dependencies rather than definitive causal effects. Although temporal and biological constraints were imposed, the resulting DAG should be interpreted as a structured representation of plausible dependency pathways and considered alongside regression, decomposition, and GSEM findings.

Sources: [2-5]

Appendix E. Generalized structural equation models

Paths selected from the network analysis were quantified using generalized structural equation models. Continuous outcomes used Gaussian identity-link models, binary outcomes used logistic models, and the cognitive outcome used a Weibull proportional-hazards specification with attained age as the time scale. Continuous age, SES, HEI-2015, and frailty were retained in the structural models. Full equation-level results are provided in Supplementary Datasheet 6.

These network-derived pathways were then carried forward into ABN-informed structural models.

Generalized structural equation models were used to quantify the pathways identified by additive Bayesian networks. For continuous nodes such as frailty score, a linear Gaussian specification was used; for categorical or ordinal nodes, appropriate generalized links were used; and for time-to-event cognitive outcomes, a Weibull survival specification was applied. A simplified two-equation system can be written as $Frailty_i = \alpha_0 + \alpha_1 HEI_i + X_i^{\top}\alpha + \epsilon_i$and $\log h_i(t) = \beta_0 + \beta_1 Frailty_i + \beta_2 HEI_i + X_i^{\top}\beta$. Under a Weibull model, the hazard function is $h_i(t) = \lambda p t^{p-1} \exp(\eta_i)$, where p is the shape parameter, λ is the scale parameter, and η_i is the linear predictor. Example Stata code is:

```
gsem (fried_sum <- zhei2015_total_score age_2016 female ses_index, family(gaussian)) ///
    (fail_cog <- fried_sum zhei2015_total_score age_2016 female ses_index, ///
     family(weibull, failure(_d) time(_t)))
```

The ABN-informed GSEM estimates for the final outcome are presented as hazard ratios with 95% confidence intervals after exponentiating log-hazard coefficients. For binary outcomes, log-odds coefficients were similarly exponentiated and presented as odds ratios with 95% confidence intervals.

Sources: [6]

Appendix F. Competing-risk and multistate sensitivity analyses

Competing Risk Regression

Competing-risk regression accounted for death as a competing event for incident cognitive impairment outcomes, including composite CIND/probable dementia, CIND-only, and probable-dementia-only endpoints. Individuals who died during follow-up were retained in the risk set with appropriate weighting rather than treated as simple censoring events, allowing estimation of subdistribution hazard ratios. Attained age was used as the underlying time scale with delayed entry at baseline. Event times were approximated as the midpoint between the last cognitively normal assessment and the first assessment showing impairment. Death dates were derived from linked mortality records, and administrative censoring occurred at the end of follow-up or the last eligible observation. Models were estimated sequentially with the same demographic, socioeconomic, behavioral, and clinical covariate blocks used in the main analyses.

Multistate Models

Multistate models characterized transitions among normal cognition, CIND, probable dementia, and death, including transitions from normal cognition to CIND, CIND to probable dementia, and each living state to death. Transition times were constructed from derived dates for incident CIND, probable dementia, and death and converted to attained age to match the primary time scale. Participants contributed person-time to multiple risk sets according to their observed state histories.

A prespecified transition matrix defined the allowable transitions, including direct transitions from normal cognition to probable dementia and from any living state to death. Transition-specific Weibull models were estimated using the same sequential covariate blocks as the main analyses. Models were not estimated for transitions with insufficient events, and robust standard errors accounted for repeated records within participants.

Interpretation

Together, these approaches provided complementary perspectives on cognitive aging. Competing-risk models estimated associations in the presence of death as a competing event, whereas multistate models characterized the sequence of cognitive-state transitions. In both approaches, frailty–cognition associations observed in minimally adjusted models attenuated after full adjustment, and HEI-2015 was not independently associated with cognitive transitions. This pattern is consistent with frailty reflecting a broader vulnerability phenotype embedded within sociodemographic and health-related factors rather than a stable independent predictor of cognitive impairment.

Key findings

Across competing-risk and multistate analyses, associations between frailty and cognitive transitions attenuated with sequential adjustment, whereas HEI-2015 was not independently associated with the outcomes. In competing-risk models, frailty was associated with higher subdistribution hazards of incident cognitive impairment in Model 1, but estimates were reduced in Model 2 and largely attenuated in the fully adjusted Model 3. Pre-frailty showed weaker and generally nonsignificant associations. These changes are consistent with confounding by measured sociodemographic, behavioral, and clinical factors.

Multistate models yielded similar patterns: frailty was associated with higher hazards of transition from normal cognition to impairment in Model 1, but estimates weakened and became nonsignificant in Models 2 and 3. No clear associations were observed for transitions to probable dementia or death after full adjustment, and HEI-2015 showed no meaningful associations across transitions. Detailed output is provided on GitHub and in Supplementary Datasheet 7.

Sources: [7, 8]

Appendix G. Reduced network sensitivity analysis

A reduced ABN and corresponding GSEM retained demographic and socioeconomic variables, HEI-2015 total score, frailty, time indicators, and cognitive outcome. This analysis assessed whether the principal network relationships persisted after exclusion of lifestyle and clinical variables. Results are provided in Supplementary Datasheet 8.

Key findings from sensitivity analysis

In the reduced ABN model for the CIND/probable-dementia outcome, the cognitive survival node retained age, non-Hispanic Black race (NHB), and frailty (fried_sum) as direct parents, along with the time indicators (t2 and t3). Age and NHB had positive associations with the outcome, and frailty was also positively associated, although its coefficient was smaller than that for NHB. These directed edges represent conditional dependencies under the specified network constraints rather than causal effects.

Within the reduced network, lower socioeconomic status (SES) was conditionally associated with minority race/ethnicity and female sex. SES was positively associated with marital status and HEI-2015, and HEI-2015 was inversely associated with frailty, consistent with lower frailty burden at higher diet quality.

Frailty was conditionally associated with age, sex, SES, and HEI-2015, placing it as an intermediate network node connecting demographic, socioeconomic, and dietary variables. These edges do not establish causal influence.

Overall, the reduced model retained a direct conditional-dependence edge between frailty and cognitive impairment while also linking frailty to upstream socioeconomic and dietary factors. HEI-2015 was not directly connected to the cognitive outcome in this specification; any pathway involving frailty should therefore be regarded as hypothesis-generating rather than evidence of causal mediation.

Corresponding reduced-GSEM estimates for the combined CIND/probable-dementia outcome are presented in Supplementary Datasheet 8.

Appendix H. Reproducibility and supporting evidence

Data management and statistical analyses were conducted in Stata 19. Network analyses were conducted in R using the abn workflow supplied with the study files. The analysis programs document sample construction, variable coding, survey settings, outcome dates, regression models, decomposition, network estimation, GSEM, and sensitivity analyses. The supplementary datasheets provide the dietary codebook and complete available numerical output.

The observational design, single dietary assessment, interval between diet and frailty measurement, complete-case selection, and limited number of probable-dementia events should be considered when interpreting the

results. Exploratory food-item, decomposition, and network analyses are intended to characterize patterns and do not establish causal effects.

Supporting evidence

Supplementary studies indicate that global diet-quality indices may not fully capture food- or component-specific associations with cognitive aging (Supplementary Datasheet 9). Although HEI-2015 has shown selected associations with dementia risk (PMID: 33044177), Mediterranean-style dietary patterns have been more consistently associated with cognitive performance (PMIDs: 35967772, 41267917, 36855023). Evidence for individual dietary components is heterogeneous. Flavonoid-rich diets and berry intake have been associated with slower cognitive decline, whereas ultra-processed foods, red meat, and artificially sweetened beverages have been associated with less favorable cognitive outcomes (PMIDs: 39292460, 22535616, 36469335, 39813835, 28428346).

Supplementary studies also characterize frailty as a broad vulnerability phenotype. Higher frailty burden has been associated with poorer cognitive performance (PMIDs: 39301115, 37903474), while dietary-pattern alignment and dietary diversity have been associated with frailty or cognitive frailty (PMIDs: 35551593, 29322507, 37686817). Frailty may also reflect inflammatory and musculoskeletal pathways. Pro-inflammatory dietary patterns have been associated with greater frailty, whereas plant-protein intake has been associated with lower frailty risk than animal-protein intake (PMIDs: 39133488, 39689376, 40672482, 35318829, 35886571). Frailty has also been associated with mortality and, in some studies, predicted mortality more strongly than cognitive status (PMIDs: 39987017, 26052349, 30468258, 27903307).

Reproducibility

For reproducibility, analyses were conducted in Stata 19 and R 4.5.3. Code was organized from data cleaning and variable derivation through regression, four-way decomposition, network learning, and structural modeling. The representative commands above illustrate key analytic steps and should be interpreted alongside the full study-specific do-files.

Supplementary references

1. Discacciati A, Bellavia A, Lee JJ, Mazumdar M, Valeri L: **Med4way: a Stata command to investigate mediating and interactive mechanisms using the four-way effect decomposition**. *Int J Epidemiol* 2018.
2. Beydoun MA, Noren Hooten N, Asefa NG, Georgescu MF, Song M, Beydoun HA, Banerjee S, Khubchandani J, Meirelles O, Launer LJ *et al*: **Telomere Length, Epigenetic Age Acceleration, and Mortality Risk in US Adult Populations: An Additive Bayesian Network Analysis**. *Aging Cell* 2025, **24**(9):e70159.
3. Beydoun MA, Weiss J, Georgescu MF, Ashe J, Maino Vieytes CA, Huang T, Beydoun HA, Noren Hooten N, Turney IC, Evans MK et al: Socio-environmental and health-related factors and their association with longitudinal change in brain neuroimaging markers through the plasma metabolome among UK adults: An additive Bayesian network analysis. Brain Behav Immun Health 2025, 50:101152.
4. Comin A, Jeremiasson A, Kratzer G, Keeling L: **Revealing the structure of the associations between housing system, facilities, management and welfare of commercial laying hens using Additive Bayesian Networks**. *Prev Vet Med* 2019, **164**:23–32.
5. **Additive Bayesian Network Modeling with the R Package abn** [https://www.jstatsoft.org/article/view/v105i08]
6. StataCorp.: **Stata Structural Equation Modeling Reference Manual: Release 18.** College Station, TX: Stata Press; 2023.

7. Austin PC, Lee DS, Fine JP: **Introduction to the Analysis of Survival Data in the Presence of Competing Risks**. *Circulation* 2016, **133**(6):601–609.

8. Putter H, Fiocco M, Geskus RB: **Tutorial in biostatistics: competing risks and multi-state models**. *Stat Med* 2007, **26**(11):2389–2430.
