# Supplementary material for: Prospective associations between diet quality, frailty and incident cognitive impairment in the health and retirement study
Source: BMC Glob Public Health. 2026 Nov 11;4:109. doi: 10.1186/s44263-026-00347-x (PMC13753220; doi:10.1186/s44263-026-00347-x)
Supplement: Supplementary file 2 [file 44263_2026_347_MOESM2_ESM.pdf]

FIGURE S1

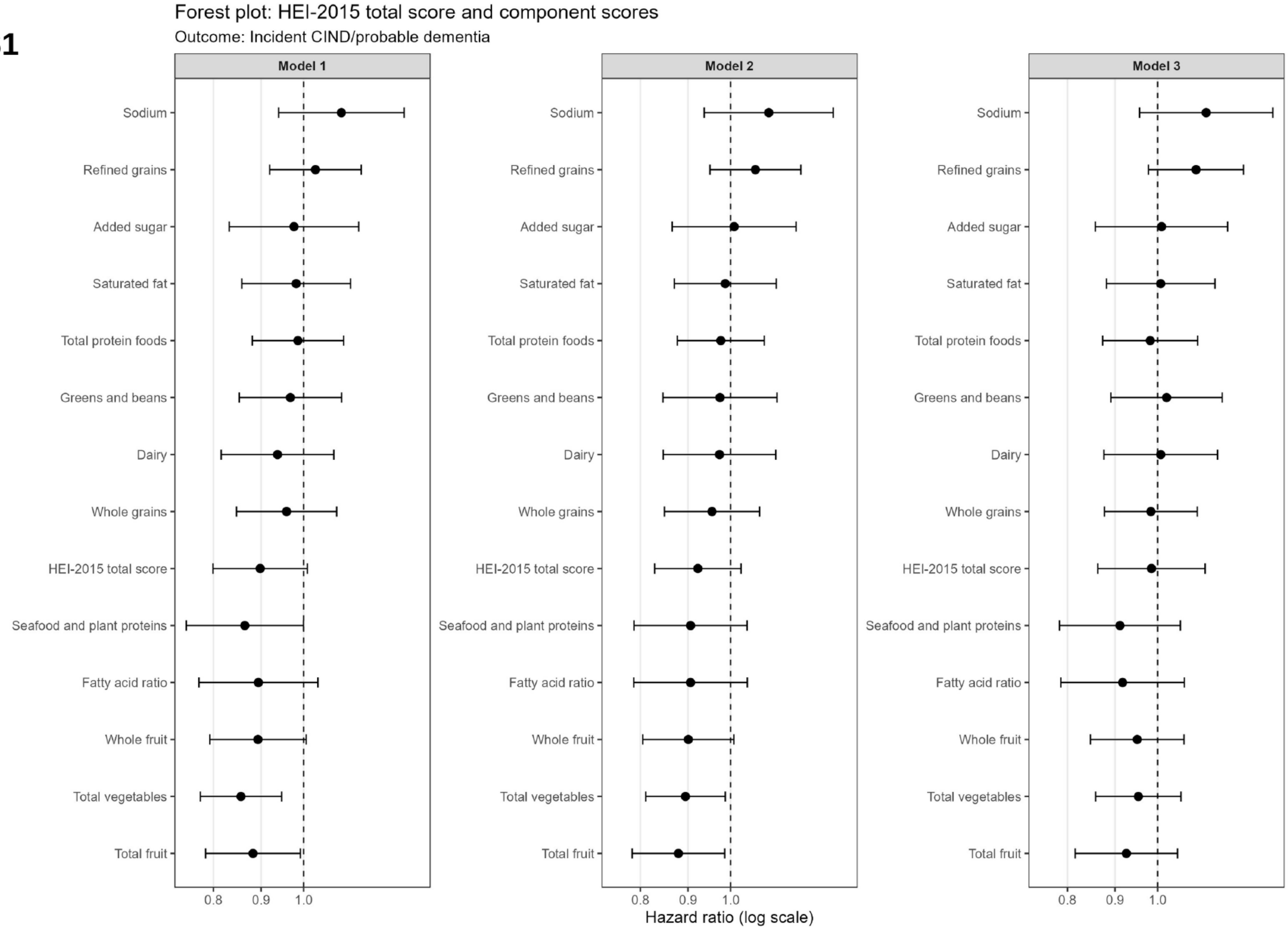
Forest plot: HEI-2015 total score and component scores
Outcome: Incident CIND/probable dementia
Model 1
Model 2
Model 3
Sodium
Refined grains
Added sugar
Saturated fat
Total protein foods
Greens and beans
Dairy
Whole grains
HEI-2015 total score
Seafood and plant proteins
Fatty acid ratio
Whole fruit
Total vegetables
Total fruit
0.8
0.9
1.0
Hazard ratio (log scale)

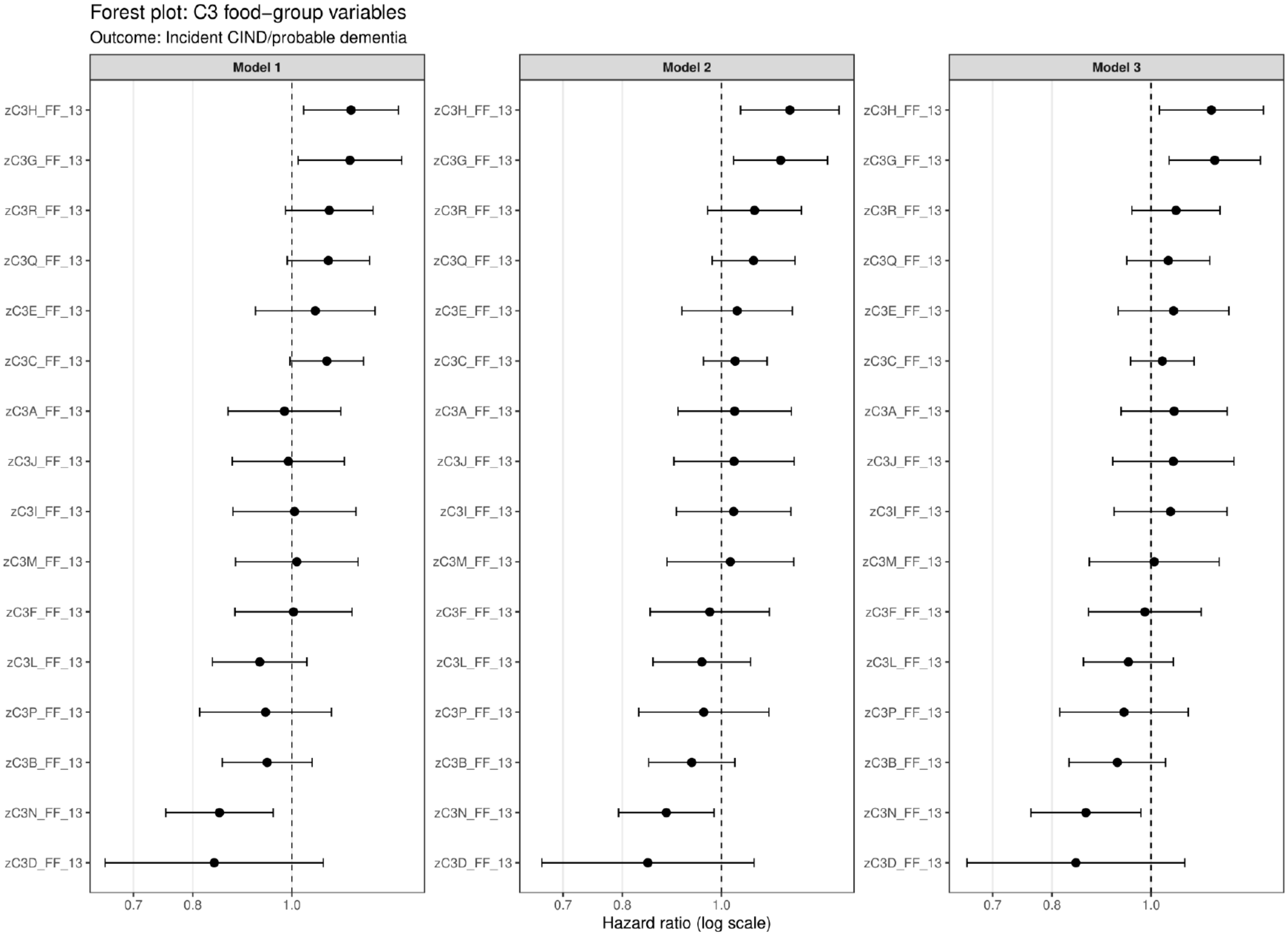
Forest plot: C3 food−group variables
Outcome: Incident CIND/probable dementia
Model 1
Model 2
Model 3
zC3H_FF_13
zC3G_FF_13
zC3R_FF_13
zC3Q_FF_13
zC3E_FF_13
zC3C_FF_13
zC3A_FF_13
zC3J_FF_13
zC3I_FF_13
zC3M_FF_13
zC3F_FF_13
zC3L_FF_13
zC3P_FF_13
zC3B_FF_13
zC3N_FF_13
zC3D_FF_13
0.7
0.8
1.0
Hazard ratio (log scale)

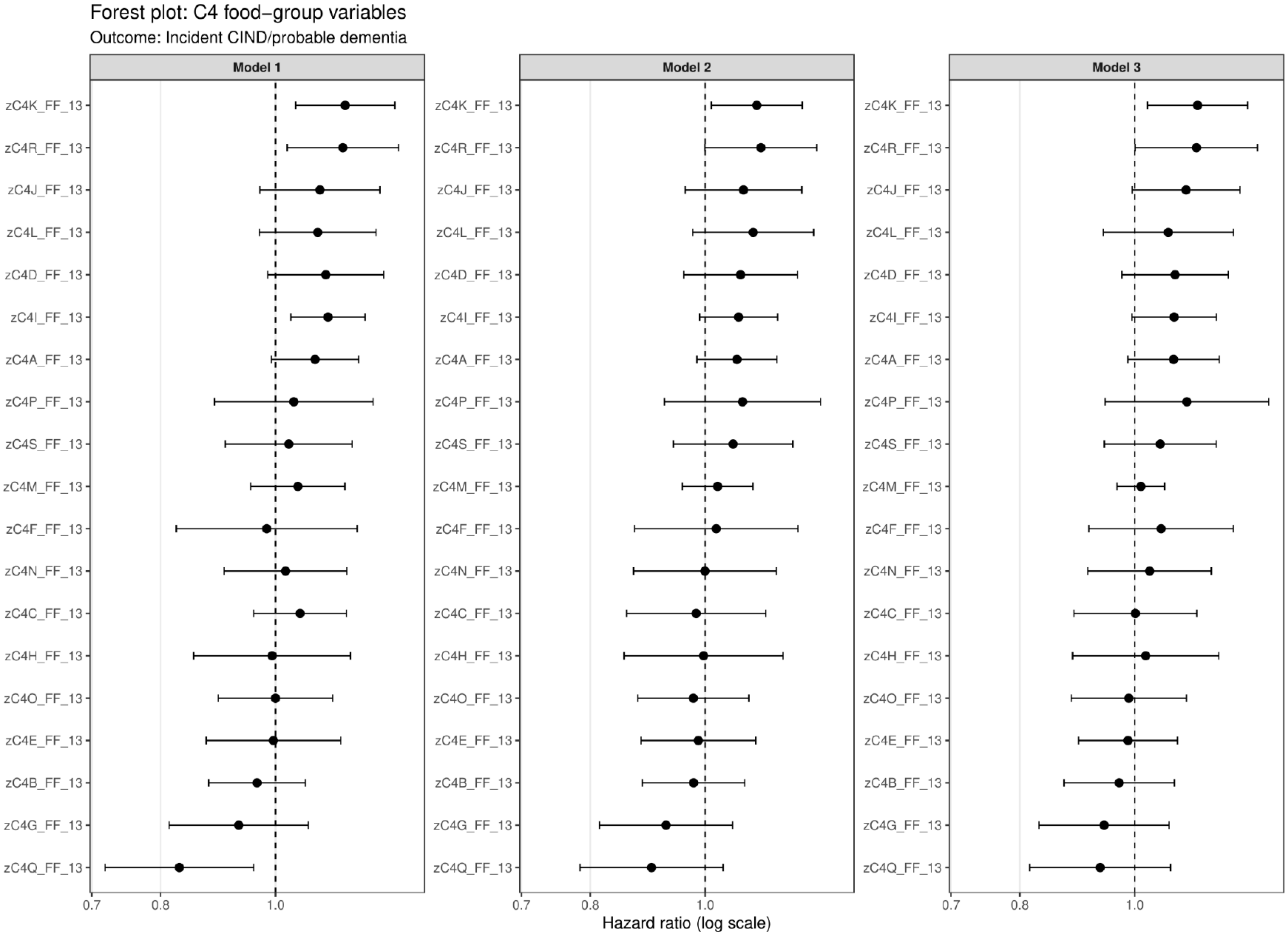

Forest plot: C4 food−group variables
Outcome: Incident CIND/probable dementia
Model 1
Model 2
Model 3
zC4K_FF_13
zC4R_FF_13
zC4J_FF_13
zC4L_FF_13
zC4D_FF_13
zC4I_FF_13
zC4A_FF_13
zC4P_FF_13
zC4S_FF_13
zC4M_FF_13
zC4F_FF_13
zC4N_FF_13
zC4C_FF_13
zC4H_FF_13
zC4O_FF_13
zC4E_FF_13
zC4B_FF_13
zC4G_FF_13
zC4Q_FF_13
0.7
0.8
1.0
Hazard ratio (log scale)

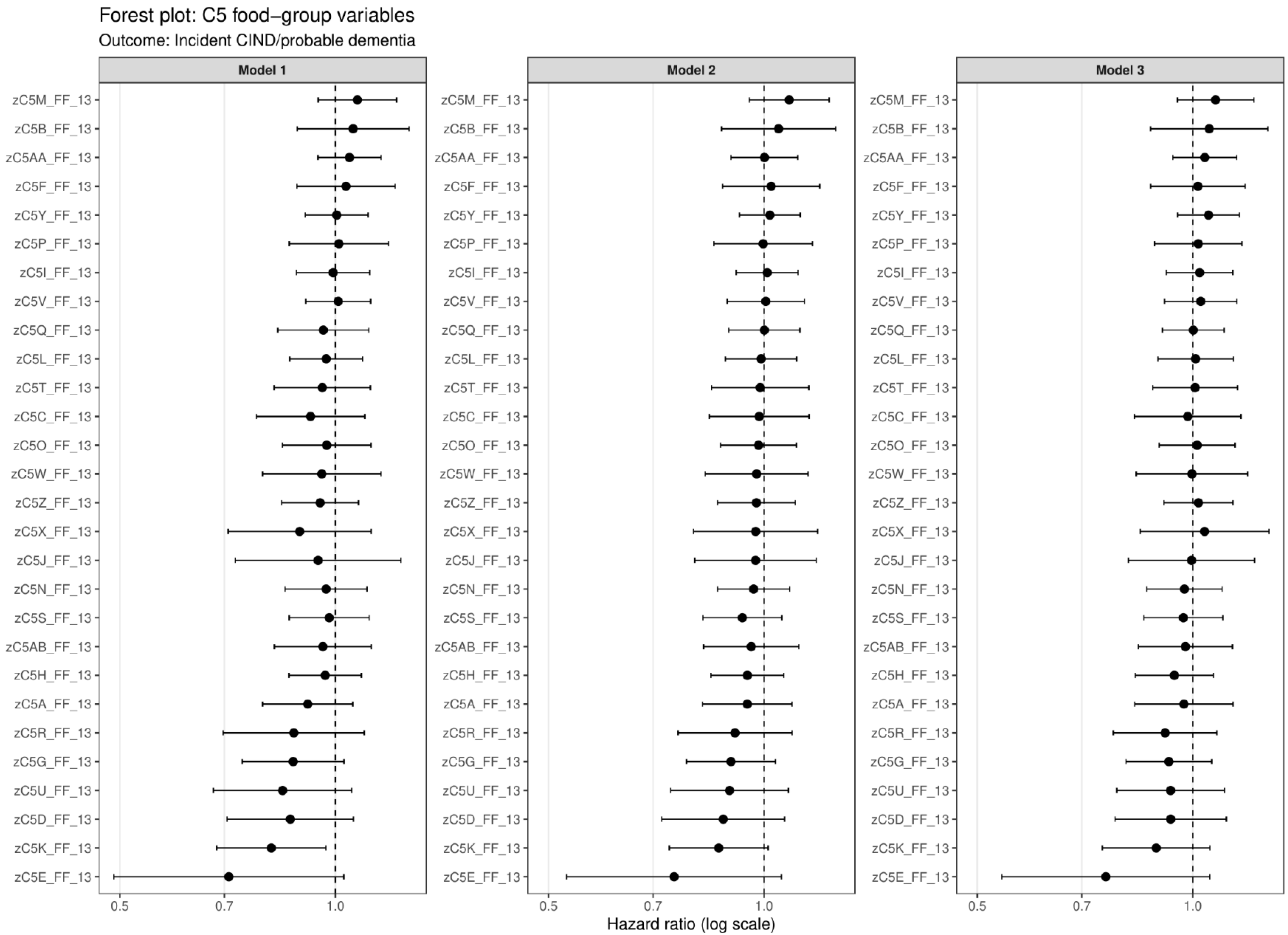
Forest plot: C5 food−group variables
Outcome: Incident CIND/probable dementia
Model 1
Model 2
Model 3
zC5M_FF_13
zC5B_FF_13
zC5AA_FF_13
zC5F_FF_13
zC5Y_FF_13
zC5P_FF_13
zC5I_FF_13
zC5V_FF_13
zC5Q_FF_13
zC5L_FF_13
zC5T_FF_13
zC5C_FF_13
zC5O_FF_13
zC5W_FF_13
zC5Z_FF_13
zC5X_FF_13
zC5J_FF_13
zC5N_FF_13
zC5S_FF_13
zC5AB_FF_13
zC5H_FF_13
zC5A_FF_13
zC5R_FF_13
zC5G_FF_13
zC5U_FF_13
zC5D_FF_13
zC5K_FF_13
zC5E_FF_13
0.5
0.7
1.0
Hazard ratio (log scale)

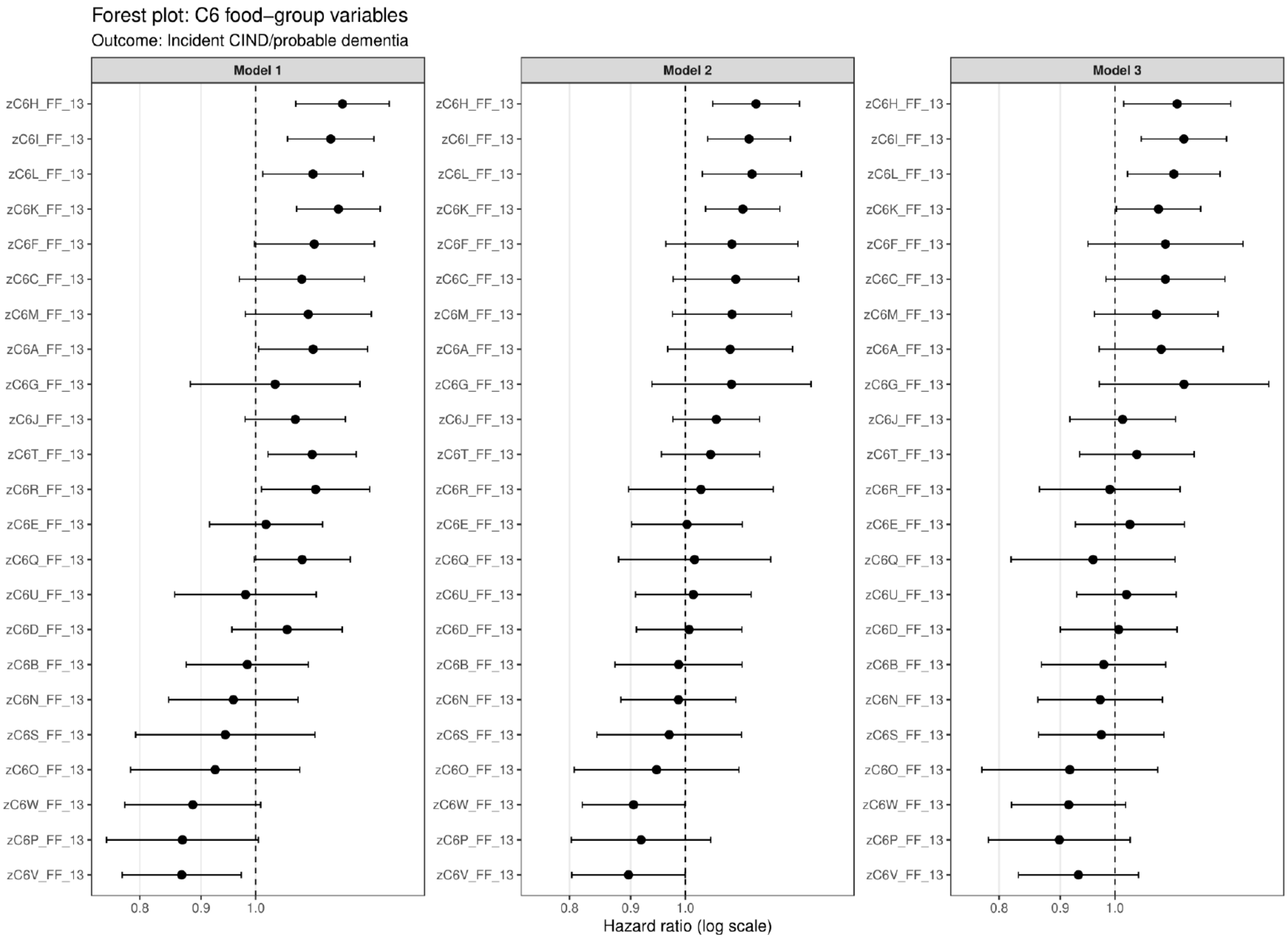

Forest plot: C6 food–group variables
Outcome: Incident CIND/probable dementia
Model 1
Model 2
Model 3
zC6H_FF_13
zC6I_FF_13
zC6L_FF_13
zC6K_FF_13
zC6F_FF_13
zC6C_FF_13
zC6M_FF_13
zC6A_FF_13
zC6G_FF_13
zC6J_FF_13
zC6T_FF_13
zC6R_FF_13
zC6E_FF_13
zC6Q_FF_13
zC6U_FF_13
zC6D_FF_13
zC6B_FF_13
zC6N_FF_13
zC6S_FF_13
zC6O_FF_13
zC6W_FF_13
zC6P_FF_13
zC6V_FF_13
0.8
0.9
1.0
Hazard ratio (log scale)

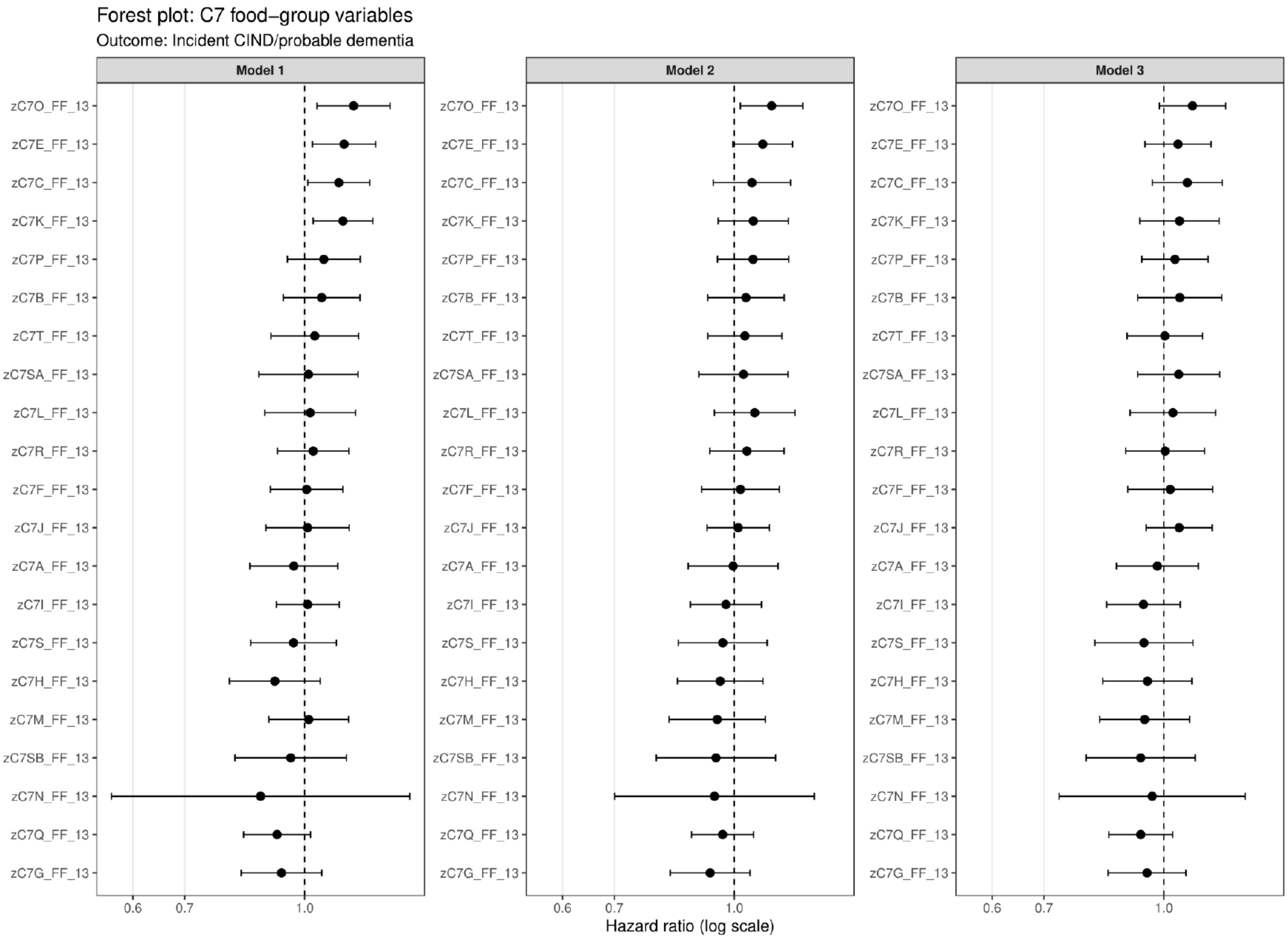
Forest plot: C7 food−group variables
Outcome: Incident CIND/probable dementia
Model 1
Model 2
Model 3
zC7O_FF_13
zC7E_FF_13
zC7C_FF_13
zC7K_FF_13
zC7P_FF_13
zC7B_FF_13
zC7T_FF_13
zC7SA_FF_13
zC7L_FF_13
zC7R_FF_13
zC7F_FF_13
zC7J_FF_13
zC7A_FF_13
zC7I_FF_13
zC7S_FF_13
zC7H_FF_13
zC7M_FF_13
zC7SB_FF_13
zC7N_FF_13
zC7Q_FF_13
zC7G_FF_13
0.6
0.7
1.0
Hazard ratio (log scale)

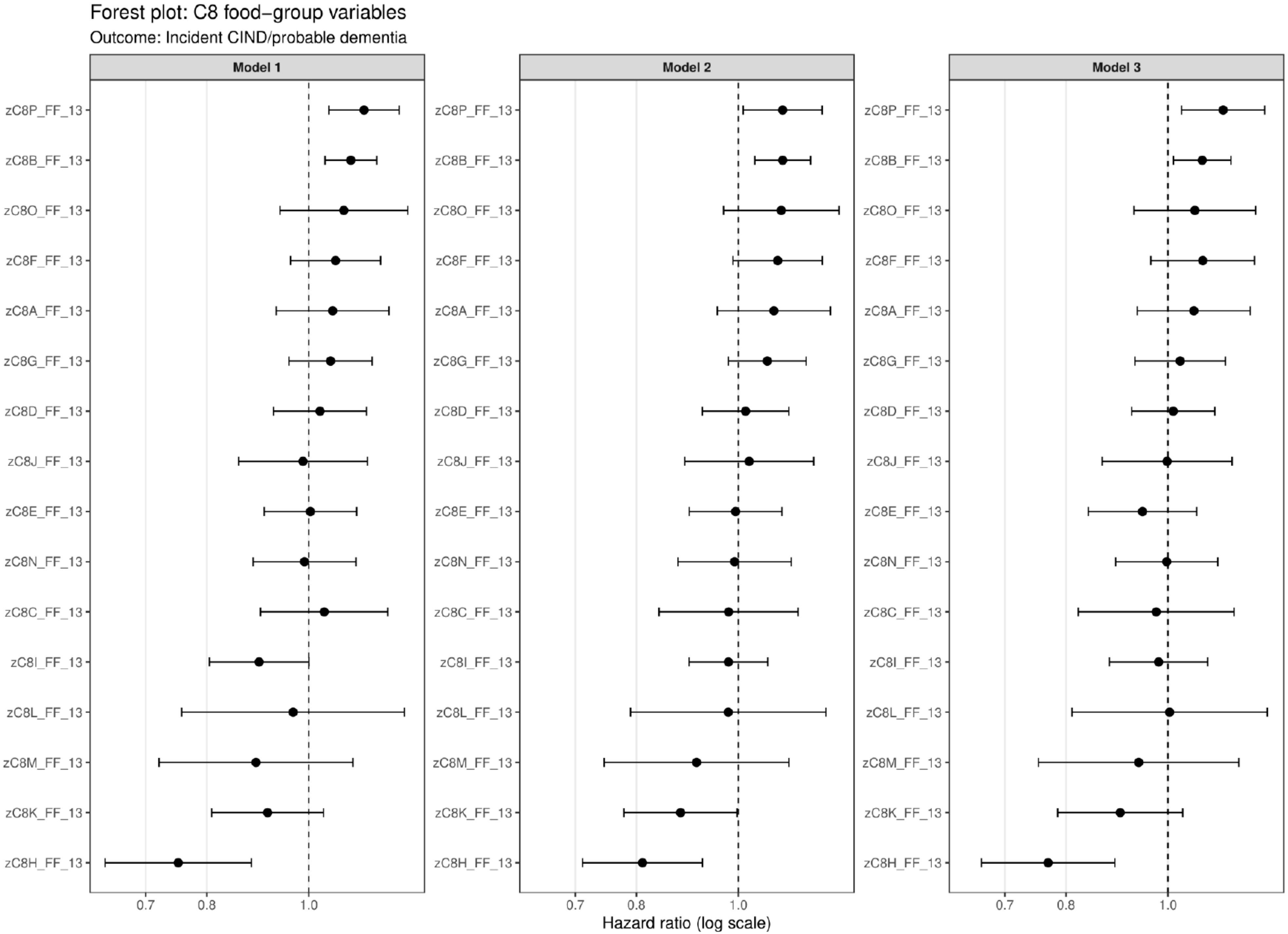
Forest plot: C8 food−group variables
Outcome: Incident CIND/probable dementia
Model 1
Model 2
Model 3
zC8P_FF_13
zC8B_FF_13
zC8O_FF_13
zC8F_FF_13
zC8A_FF_13
zC8G_FF_13
zC8D_FF_13
zC8J_FF_13
zC8E_FF_13
zC8N_FF_13
zC8C_FF_13
zC8I_FF_13
zC8L_FF_13
zC8M_FF_13
zC8K_FF_13
zC8H_FF_13
0.7
0.8
1.0
Hazard ratio (log scale)

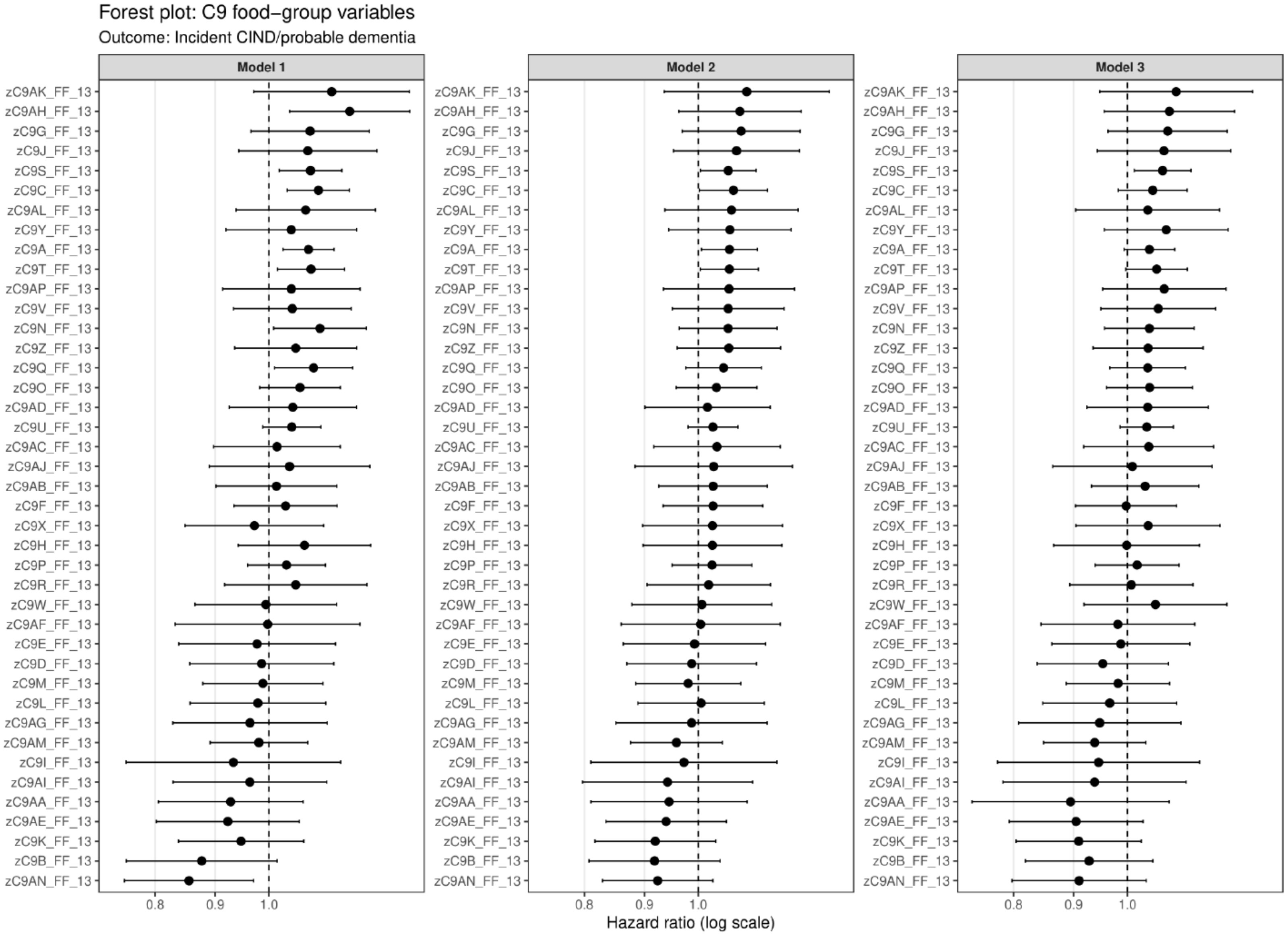

Forest plot: C9 food−group variables
Outcome: Incident CIND/probable dementia
Model 1
Model 2
Model 3
zC9AK_FF_13
zC9AH_FF_13
zC9G_FF_13
zC9J_FF_13
zC9S_FF_13
zC9C_FF_13
zC9AL_FF_13
zC9Y_FF_13
zC9A_FF_13
zC9T_FF_13
zC9AP_FF_13
zC9V_FF_13
zC9N_FF_13
zC9Z_FF_13
zC9Q_FF_13
zC9O_FF_13
zC9AD_FF_13
zC9U_FF_13
zC9AC_FF_13
zC9AJ_FF_13
zC9AB_FF_13
zC9F_FF_13
zC9X_FF_13
zC9H_FF_13
zC9P_FF_13
zC9R_FF_13
zC9W_FF_13
zC9AF_FF_13
zC9E_FF_13
zC9D_FF_13
zC9M_FF_13
zC9L_FF_13
zC9AG_FF_13
zC9AM_FF_13
zC9I_FF_13
zC9AI_FF_13
zC9AA_FF_13
zC9AE_FF_13
zC9K_FF_13
zC9B_FF_13
zC9AN_FF_13
0.8
0.9
1.0
Hazard ratio (log scale)

Figure S1. Forest plots of HEI-2015 components and food groups with incident cognitive impairment: Full set of results

Forest plots display fully adjusted hazard ratios for associations between standardized HEI-2015 components and food-group variables with incident cognitive impairment (CIND/dementia composite), based on survey-weighted Cox models. Dietary exposures were derived from the 2013 Health Care and Nutrition Study and grouped according to the dietary codebook. HEI-2015 components represent adherence to dietary guidelines across adequacy domains (e.g., total fruit, whole fruit, total vegetables, greens and beans, whole grains, dairy, total protein foods, seafood and plant proteins, fatty acid ratio) and moderation domains (e.g., refined grains, sodium, added sugars, saturated fats).

Food-group variables (C3–C9 series) correspond to major dietary domains: C3 (dairy, fats, spreads), C4 (fruits and juices), C5 (vegetables, legumes, soy), C6 (protein foods including meat, eggs, seafood), C7 (grains, breads, and starchy foods), C8 (beverages and alcohol), and C9 (sweets, snacks, and condiments). Hazard ratios are presented per 1-standard deviation increase in intake, with horizontal lines representing 95% confidence intervals. Values <1 indicate inverse associations (lower risk), whereas values >1 indicate positive associations (higher risk).

Abbreviations: CI = confidence interval; HEI-2015 = Healthy Eating Index–2015; HR = hazard ratio; SD = standard deviation.

FIGURE S2

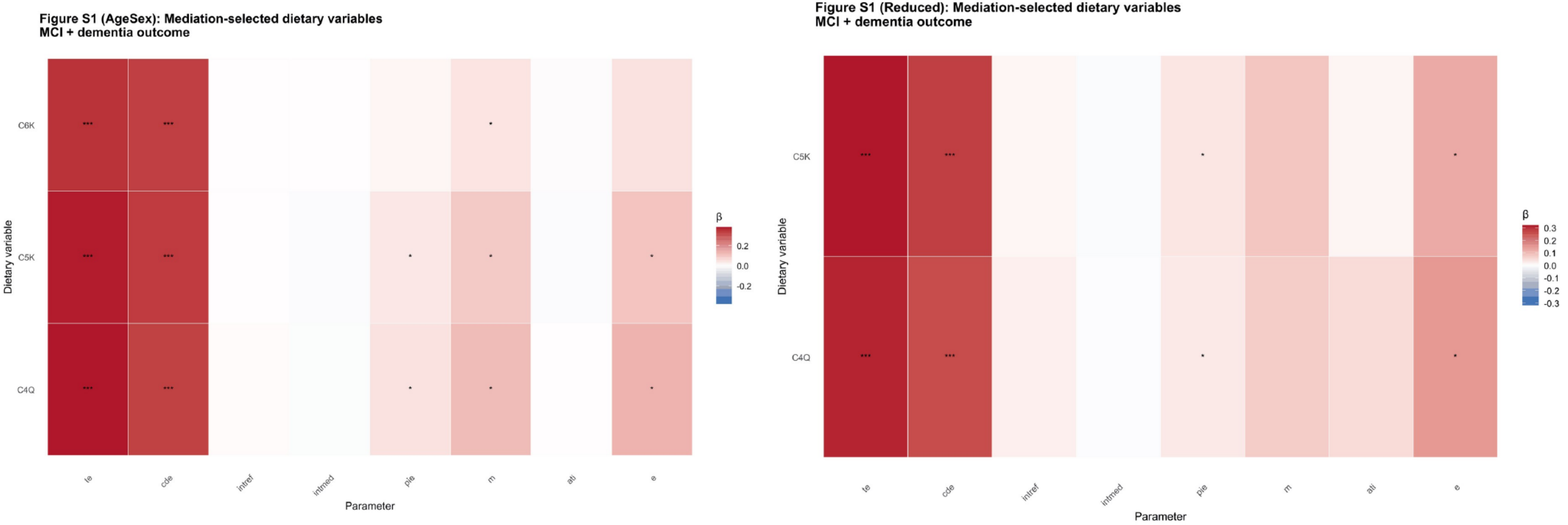
Figure S1 (AgeSex): Mediation-selected dietary variables
MCI + dementia outcome
Dietary variable
C6K
C5K
C4Q
te
cde
intref
intmed
pie
m
ati
e
Parameter
β
0.2
0.0
-0.2
Figure S1 (Reduced): Mediation-selected dietary variables
MCI + dementia outcome
Dietary variable
C5K
C4Q
te
cde
intref
intmed
pie
m
ati
e
Parameter
β
0.3
0.2
0.1
0.0
-0.1
-0.2
-0.3

Figure S2. Heatmap of four-way decomposition results highlighting mediation components

- Heatmap summarizing selected four-way decomposition analyses focusing on mediation pathways linking frailty (exposure), diet (HEI-2015 total score or dietary components), and incident cognitive impairment in the HRS (2016–2022).
- Rows represent dietary exposures (HEI-2015 total score and food-group variables), restricted to those with at least one statistically significant mediation-related component. Columns display four-way decomposition parameters derived from the med4way approach, including controlled direct effect (CDE), pure indirect effect (PIE), mediated interaction (INTmed), reference interaction (INTref), total effect (TE), and mediation-related proportion measures (e.g., proportion mediated, overall mediated effect = PIE + INTmed).
- Color gradients represent the magnitude and direction of effects (red = higher risk; blue = lower risk), with numerical values shown in each cell and statistical significance indicated by asterisks. The visualization enables comparison of mediation contributions across dietary variables.
- This figure highlights variables with statistically significant mediation components (PIE, INTmed, or overall mediated effect), reflecting the extent to which diet lies on the pathway between frailty and cognitive impairment. The pure indirect effect (PIE) represents mediation independent of interaction, while INTmed captures mediation that depends on exposure–mediator interaction.
- Across analyses, mediation effects are generally small and often not statistically significant, consistent with primary results indicating that diet contributes minimally to the frailty–cognition association through indirect pathways. Instead, most of the total effect is explained by the controlled direct effect of frailty, reinforcing its role as a proximal determinant of cognitive impairment.
- All models incorporate survey weights and adjust for demographic, socioeconomic, behavioral, and clinical covariates. Estimates are derived from Cox proportional hazards models for time-to-event outcomes and linear models for mediators, consistent with counterfactual decomposition assumptions.

FIGURE S3

(A) Multistate transition boxes

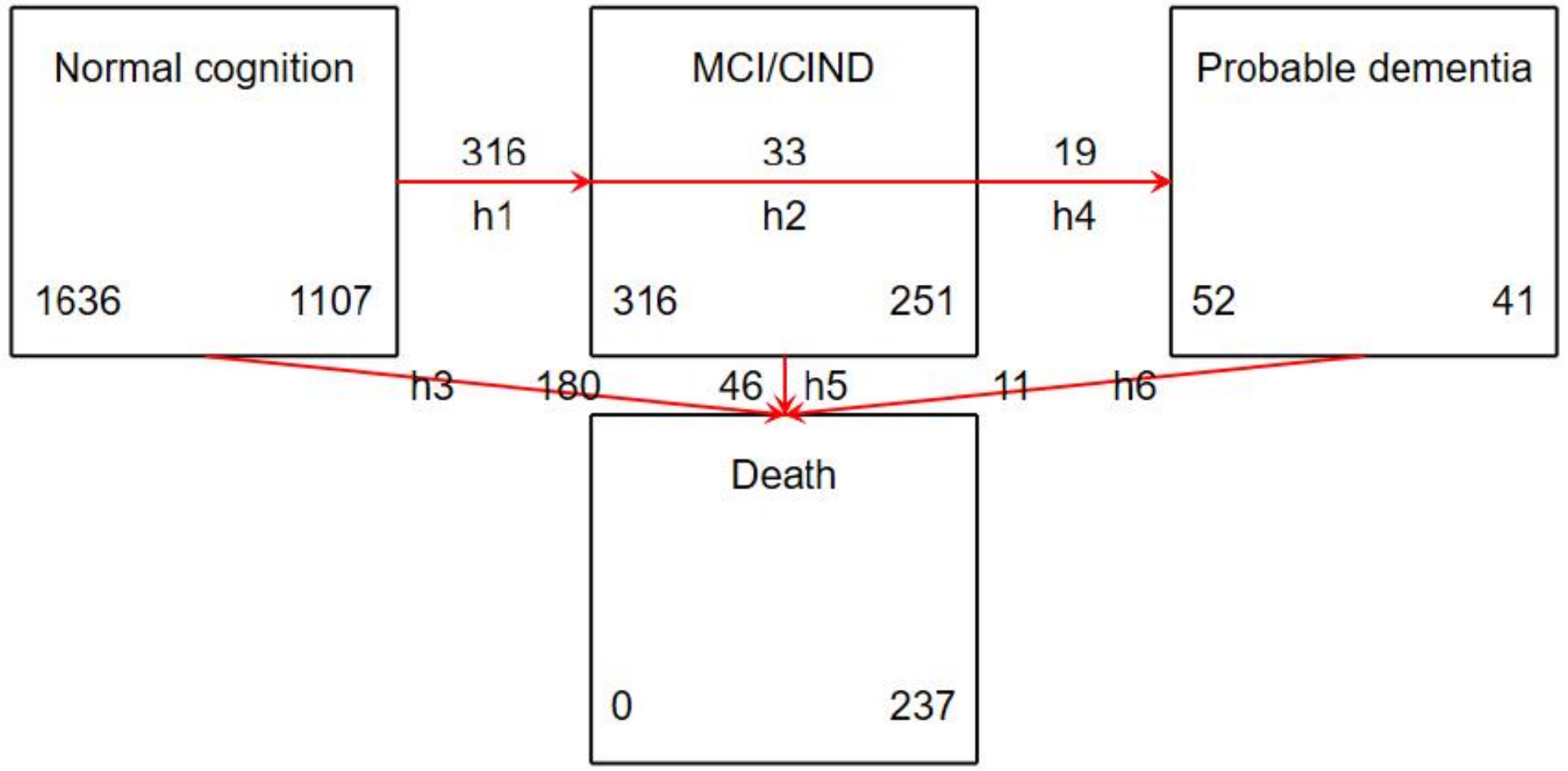

(B) Multistate transition probabilities

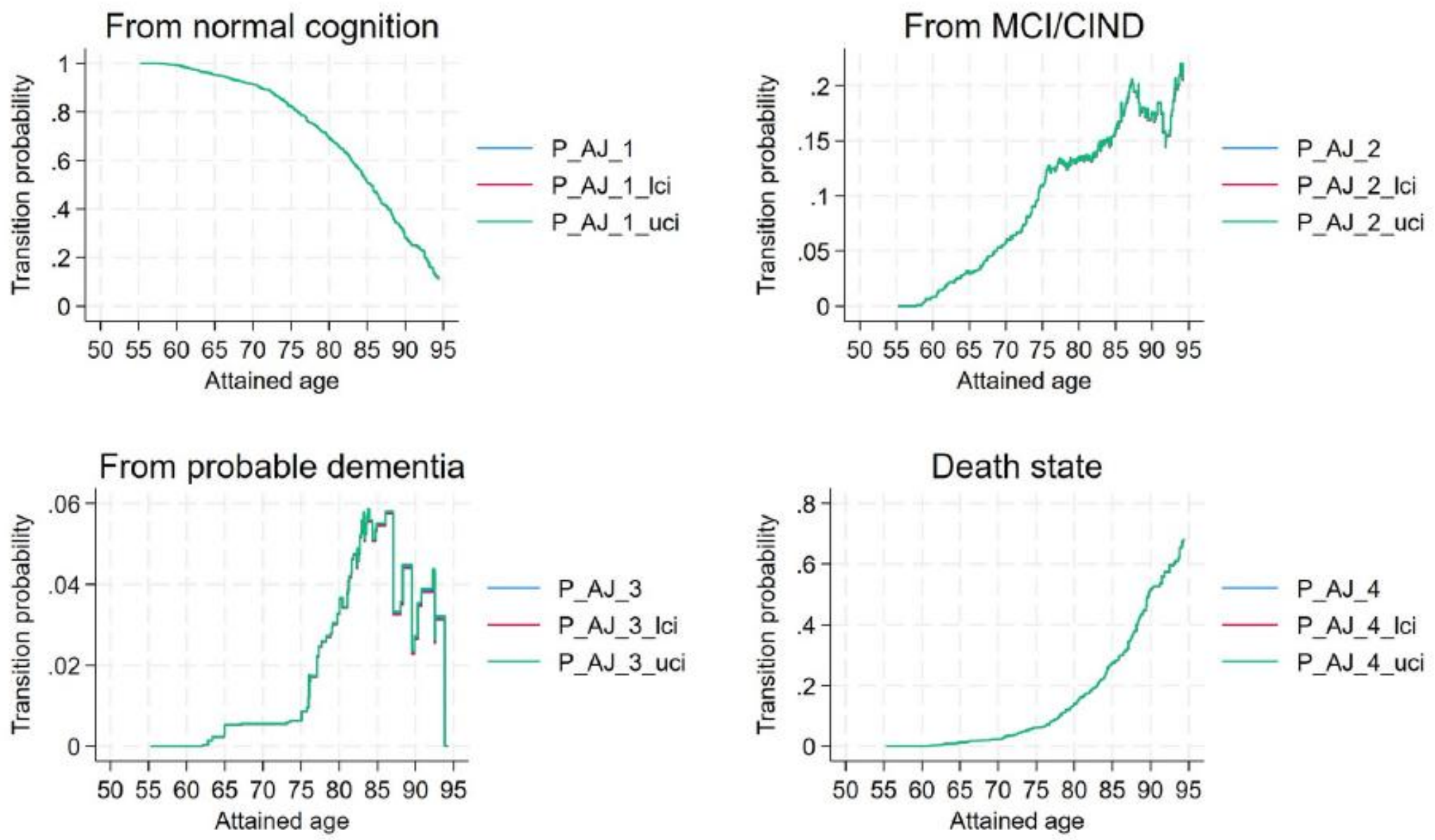

(C) Predicted survival probabilities from Weibull models for 3 transitions

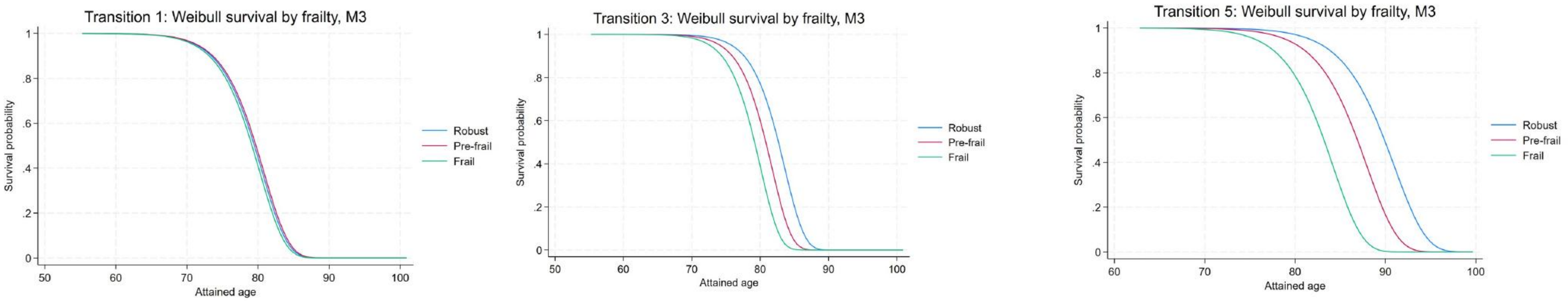

Figure S3. Multistate model findings

Footnote: Multistate models evaluated transitions across cognitive states (normal → CIND → dementia → death) using Weibull regression with transition-specific hazard estimation, conditional on sufficient events. Competing-risk models accounted for death as a competing event. Predicted survival probabilities were derived for key transitions, illustrating attenuation of frailty–cognition associations after full adjustment, while maintaining strong associations with mortality transitions.

FIGURE S4

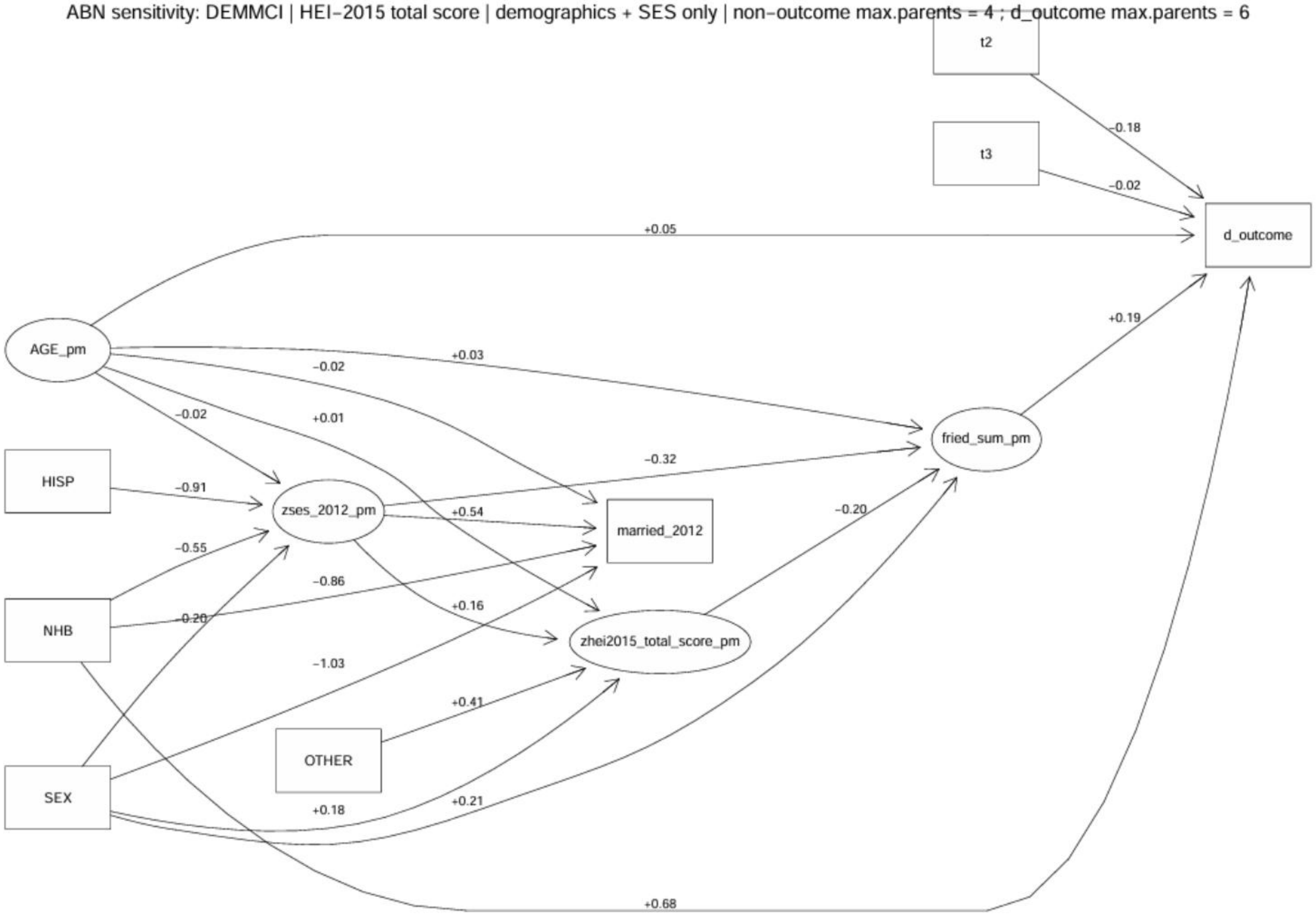
ABN sensitivity: DEMMCI | HEI−2015 total score | demographics + SES only | non−outcome max.parents = 4 ; d_outcome max.parents = 6
t2
t3
d_outcome
−0.18
−0.02
+0.05
+0.19
AGE_pm
+0.03
−0.02
−0.02
+0.01
fried_sum_pm
HISP
−0.32
−0.91
zses_2012_pm
+0.54
married_2012
−0.20
−0.55
NHB
−0.86
+0.16
−0.20
zhei2015_total_score_pm
−1.03
+0.41
OTHER
SEX
+0.18
+0.21
+0.68

Figure S4. Reduced Additive Bayesian Networks (ABN) findings

Footnote: Reduced additive Bayesian network (ABN) structures informed generalized structural equation models (GSEM), confirming frailty as an upstream determinant with modest direct effects sensitive to model specification. Together, these sensitivity analyses support robustness of the primary findings.
