# Supplementary material for: Prospective associations between diet quality, frailty and incident cognitive impairment in the health and retirement study
Source: BMC Glob Public Health. 2026 Nov 11;4:109. doi: 10.1186/s44263-026-00347-x (PMC13753220; doi:10.1186/s44263-026-00347-x)
Supplement: Supplementary file 3 [file 44263_2026_347_MOESM5_ESM.pdf]

LIST OF SUPPLEMENTARY MATERIALS

Supplementary Materials 1: Appendices A-H

Supplementary Materials 2: Supplementary Figures 1-4

Figure S1. Forest plots of HEI-2015 components and food groups with incident cognitive impairment: Full set of results

Figure S2. Heatmap of four-way decomposition results highlighting mediation components

Figure S3. Multistate model findings

Figure S4. Reduced Additive Bayesian Network (ABN) findings

Supplementary Materials 3: Supplementary datasheets 1-9

Supplementary datasheet 1. Dietary codebook variable index

Supplementary datasheet 2. Full set of survey-weighted multivariable linear regression results examining the association between diet quality (HEI-2015) and frailty score in the Health and Retirement Study (HRS) analytic sample (N = 1,636)

Supplementary datasheet 3. full set of survey-weighted Cox proportional hazards model results examining associations between individual dietary food groups and incident cognitive impairment in the Health and Retirement Study (HRS) analytic sample (N = 1,636).

Supplementary datasheet 4. Complete set of counterfactual-based four-way decomposition results evaluating the joint roles of frailty (exposure) and diet quality or dietary variables (mediator and effect modifier) in relation to incident cognitive impairment.

Supplementary datasheet 5. Additive Bayesian Network (ABN) Results

Supplementary datasheet 6. Full GSEM results: incident MCI/CIND + probable dementia (combined outcome)

Supplementary datasheet 7. Competing risk regression and multistate model findings

Supplementary datasheet 8. Reduced Additive Bayesian Network (ABN) and Generalized Structural Equations Modeling (GSEM) findings

Supplementary datasheet 9. Extended literature review

Supplementary Materials 4:

Survey-weighted baseline characteristics of participants by HEI-2015 categories and frailty status, Health and Retirement Study (2016)
